# Comparison of pyrolysis gas chromatography/mass spectrometry and hyperspectral FTIR imaging spectroscopy for the analysis of microplastics

**DOI:** 10.1007/s00216-020-02979-w

**Published:** 2020-10-26

**Authors:** Sebastian Primpke, Marten Fischer, Claudia Lorenz, Gunnar Gerdts, Barbara M. Scholz-Böttcher

**Affiliations:** 1 Alfred-Wegener-Institute Helmholtz Centre for Polar and Marine Research, Biologische Anstalt Helgoland, Kurpromenade 201, 27498 Helgoland, Germany; 2 Institute for Chemistry and Biology of the Marine Environment (ICBM), Carl von Ossietzky University of Oldenburg, P.O. Box 2503, 26111 Oldenburg, Germany; 3 Present Address: Department of the Built Environment, Aalborg University, Thomas Manns Vej 23, 9220 Aalborg Øst, Denmark

**Keywords:** Intercomparison, Py-GC/MS, FTIR, Spectroscopy, Mass spectrometry, Environmental samples

## Abstract

Analysis of microplastics (MP) in environmental samples is an emerging field, which is performed with various methods and instruments based either on spectroscopy or thermoanalytical methods. In general, both approaches result in two different types of data sets that are either mass or particle number related. Depending on detection limits of the respective method and instrumentation the derived polymer composition trends may vary. In this study, we compare the results of hyperspectral Fourier-transform infrared (FTIR) imaging analysis and pyrolysis gas chromatography-mass spectrometry (Py-GC/MS) analysis performed on a set of environmental samples that differ in complexity and degree of microplastic contamination. The measurements were conducted consecutively, and on exactly the same sample. First, the samples were investigated with FTIR using aluminum oxide filters; subsequently, these were crushed, transferred to glass fiber filters, in pyrolysis cups, and measured via Py-GC/MS. After a general data harmonization step, the trends in MP contamination were thoroughly investigated with regard to the respective sample set and the derived polymer compositions. While the overall trends in MP contamination were very similar, differences were observed in the polymer compositions. Furthermore, polymer masses were empirically calculated from FTIR data and compared with the Py-GC/MS results. Here, a most plausible shape-related overestimation of the calculated polymer masses was observed in samples with larger particles and increased particle numbers. Taking into account the different measurement principles of both methods, all results were examined and discussed, and future needs for harmonization of intermethodological results were identified and highlighted.

**Electronic supplementary material:**

The online version of this article (10.1007/s00216-020-02979-w) contains supplementary material, which is available to authorized users.

**Figure:**
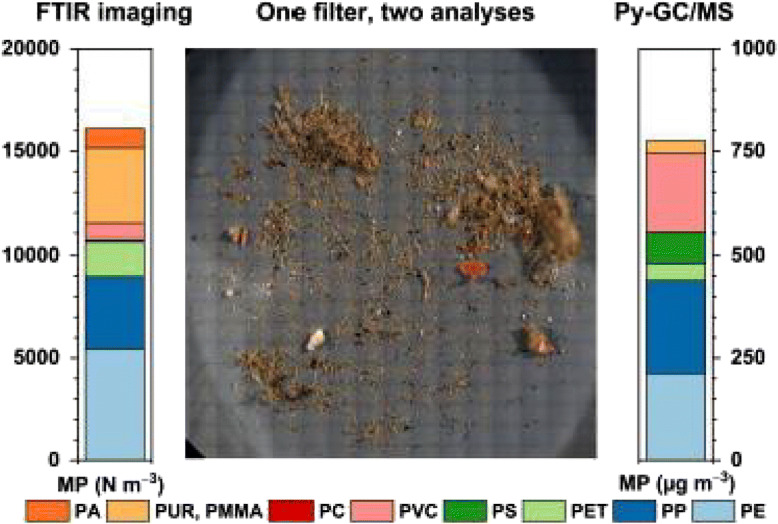
Graphical abstract

## Introduction

Microplastic (MP) particles [1] are considered as a new pollutant in the environment and their analysis is an emerging field in analytical chemistry [2–5]. The ubiquitous pollution with MP causes concern to society and governments, and first legislations to govern MP in the marine environment, as well as in consumer products, are in preparation, e.g., in California, USA [6–8].

The analysis of MP faces several challenges due to variable properties of the targeted particles, complexity of environmental samples, and associated analytical costs [2, 3, 5, 9]. Additionally, MP contamination can either be expressed as the number of particles present in a sample or as the mass concentration of the respective polymer. So far, no MP mass concentrations are related to any environmental risk assessment while in particular small MP particles and numbers are important information for assessing the environmental impact of MP inclusive human health [10–12]. At the current state, MP masses are ideal for modelling and mass balancing for, e.g., wastewater treatment plants or general load determination. Besides size and mass of particles, their chemical identity is crucial as different polymer types may act differently due to their chemical composition, absorbed or inherent chemicals and density in modelling, and risk assessment studies [13].

To determine the chemical nature of the particles in general, two analytical approaches are currently mainly used, based either on spectroscopy or thermal degradation of the polymers. In the first case, the sample is often analyzed either via Fourier-transform infrared (FTIR) [14] or Raman spectroscopy [15, 16]. Both techniques allow polymer identification by measuring the vibrations of specific molecular bonds and functionalities. The derived absorption spectra deliver a fingerprint of the material which is further analyzed via library searches against reference spectra. Both methods are currently used in many studies for the analysis of MP [2, 3, 5].

Furthermore, combined with microscopes [2, 17], both techniques allow the determination of small MP (> 10 μm for FTIR; > 1 μm for Raman) by particle-based [18–21] or imaging approaches [22–25]. Especially for FTIR, hyperspectral chemical imaging can be performed fast using focal plane array detectors [26, 27]. Currently, several approaches are available to analyze the obtained data, using spectral correlation [23, 28], selective band separation and analysis [29, 30], machine learning [31], or classifiers [32]. One of the most applied or amended approaches [3] is currently based on the automated analysis pipeline (AAP) [23] using vector-normalized spectra and a specialized database [33]. This approach has the advantage to exclude human bias nearly completely and to operate via free of charge accessible tools based on Python scripts and the software siMPle [34].

Via these tools, currently up to 32 polymer types [33] can be fully automated identified. The assigned particles are characterized (e.g., size, form factor) and quantified in a harmonized manner independent from the software and instrument [34].

In contrast to spectroscopic methods, thermoanalytical methods are destructive techniques. The sample is thermally decomposed under defined conditions, using specialized units like pyrolyzers [35] or thermogravimetric systems [36, 37]. The formed pyrolysis products are then analyzed via gas chromatography coupled with mass spectrometry (GC/MS) [38–41]. The respective polymer type is identified by its characteristic decomposition products. Thermoanalytical methods can be performed either qualitatively for single MP particles [42–48], their organic additives [49–54] and rubbers [55], but also for complex environmental samples to identify MP polymer types and their respective, simultaneous quantification. Currently, two major techniques are applied for mass-quantitative MP analysis, Pyrolysis-GC/MS (Py-GC/MS) [35, 56–63], or thermo-extraction desorption GC/MS (TED-GC/MS) [64–66], which in both cases detect and quantify the amount of MP via characteristic pyrolysis products and their respective indicator ions (see, e.g., reference [3], Table 1 for a more detailed comparison).

Some of these techniques were compared on a technical level in a publication by Elert et al. [67]. Here, FTIR, Raman, TED-GC/MS, and size exclusion chromatography were investigated with a very limited set of polymers. In contrast, Cabernard et al. [21] and Käppler et al. [24] investigated the performance of FTIR and Raman for the analysis of MP in environmental samples. For isolated particles [46–48] and fibers [48], the performance of (microscopy-supported) ATR-FTIR, Raman, and Py-GC/MS was evaluated depicting the complementary character of thermal degrading and spectroscopic techniques.

Almost all studies on small MP (< 100 μm) are performed with a single technique and prevent a profound comparison of resulting data, e.g., particle abundances vs. mass quantification. Other calculative approaches, such as the recently published mass estimation of FTIR microscopy data by Simon et al. [68], have been developed to bridge this gap, but have not been tested against Py-GC/MS measurements. To enable qualitative and quantitative MP data comparison of relevant environmental studies obtained by different methods, an analytical approach that focuses on their comparability and possible limitations is necessary to provide a starting point for harmonization of future MP analysis and a potential retrospective application.

In this study, we present a method comparison utilizing various environmental sample sets of varying complexity. In direct succession, Anodisc filters were first analyzed via FTIR imaging followed by Py-GC/MS measurements of the same crushed filter membrane. The first goal of this study was to evaluate differences and similarities in the resulting data sets, focusing on the general qualitative data comparison in order to provide solutions for their harmonization. The chosen sample sets with different MP contamination levels were part of previous FTIR studies on treated waste water [22, 69] (high level), marine sediments (medium level), and surface water (trace level) [70]. The second goal was to compare the potential of quantitative data conversion and facing calculated polymer masses [68] based on the FTIR particle counts against the direct mass quantification received by Py-GC/MS. These approaches emphasize the extent of harmonization potential between Py-GC/MS and FTIR imaging data. Furthermore, recommendations of hyperspectral FTIR imaging and Py-GC/MS for ecotoxicology studies and monitoring are derived.

## Material and methods

### Surface water and sediment samples

The sample preparation and digestions of the environmental matrix is described in full detail in Lorenz et al. [70]. The sample locations are summarized in the Electronic Supplementary Material (ESM) ESM 1.xlsx. In brief summary, the sediment samples were treated by density separation using the microplastic sediment separator (MPSS) [71] with a high density ZnCl_2_ (*ρ* = 1.75 g cm^−3^) solution. The supernatant was collected for further sample extraction. Here, the sediment and the surface water samples were size fractionated by sieving over a 500-μm stainless steel mesh (Haver & Boecker OHG). The size fraction ≤ 500 μm was treated via an enzymatic digestion [72], concentrated onto Anodisc (25 mm diameter, PP-supported, 0.2 μm pore size, GE Whatman) filters and placed onto a CaF_2_ (25 mm diameter, 2 mm in thickness, Korth Kristalle, Germany) window prior to measurement (see ESM 2.pdf paragraph S1 for the measurement details) [70].

### Treated waste water samples

The samples were originally taken in a previous study in collaboration [69, 73] with the Oldenburg-East-Frisian water board (OOWV) at two waste water treatment plants (see ESM 1.xlsx) in the regions of Oldenburg and Holdorf in Germany universally at the effluent. At Oldenburg, location samples were taken additionally at the inflow to a post-filtration unit. These additional samples were used to screen the efficiency of the filtration unit similar to Mintenig et al. [74]. The samples were treated via enzymatic digestion [72] and concentrated on Anodisc (0.2 μm) filters. During the investigation for microfibers in a previous study [22], the sample surface was covered with a BaF_2_ window during measurement. Its removal possibly caused a particle loss, and made it necessary to re-measure the samples to excluded false interpretation. For re-measurement, the filters were placed on top of a CaF_2_ window (see ESM 2.pdf paragraph S1 for measurement details) and afterwards transferred to the Py-GC/MS laboratory. The results of the re-measurement showed a similar pattern (see ESM 2.pdf Fig. S1) as found in the previous studies [22, 69] with higher concentration on the second sampling day (17 August 2015) and prior to a post-filtration unit in Oldenburg while the Blank showed barely any synthetic particles.

### Mass estimation

The Python script of AAP [23] was enhanced to allow the mass estimation following Simon et al. [68]. To achieve this, the Feret diameter (FD) and the elongation (EL) of the particles were calculated. Based on these two values, the widest and shortest length (*L*_m_) of the particle is available. Mass estimates are based on the following equations:

1 $$ {L}_{\mathrm{m}}=\frac{\mathrm{FD}}{\mathrm{EL}} $$

2 $$ {m}_{\mathrm{calc}}=\frac{4}{3}\times \pi \times \frac{\mathrm{FD}}{2}\times \frac{L_{\mathrm{m}}}{2}\times \frac{L_{\mathrm{m}}\times {R}_{\mathrm{D}}}{2}\times {\rho}_{\mathrm{polymer}} $$

To calculate the mass, the ratio (*R*_D_) between minor and major dimension of all determined MP of one sample (Holdorf1708) was calculated. A median value of 0.7 ± 0.3 was achieved, which was used for all further calculations as well as the individual densities *ρ*_polymer_ of the siMPle database [34].

### Sample transfer for Py-GC/MS measurements

For Py-GC/MS analysis, the stabilizing PP margin of Anodisc filters was removed. The punched (glass rod) out inner aluminum oxide part with the sample (see ESM 2.pdf Fig. S2) was crushed (glass rod) and concentrated on a glass fiber filter (Ø 20 mm, 1 μm pore size, Pall Life Sciences; pretreated for 12 h in a muffle furnace at 400 °C). The volume of the resulting filter cake mainly of alumina required a sample partition into two aliquots. The glass fiber filter was cut in half with a scalpel; each half was folded with tweezers and placed in a separate pyrolysis cup.

### Py-GC/MS measurements

Py-GC/MS measurements were carried out with a micro furnace pyrolyzer EGA/Py-3030D (FrontierLab, Japan) equipped with an auto-shot sampler AS-1020E (FrontierLab, Japan). The pyrolyzer was mounted to an Agilent 7890B gas chromatograph containing a deactivated retention gap connected to a DB-5MS-column. The gas chromatograph was attached to an Agilent MSD 5977A mass spectrometer. Further details are given in ESM 2.pdf Table S1.

For internal standardization of the pyrolytic process, a mixture of 50 μl of 9-tetradecyl-1,2,3,4,5,6,7,8-octahydro anthracene (0.01 μg/μl in *n*-hexane, Sigma-Aldrich), 9-dodecyl-1,2,3,4,5,6,7,8-octahydro anthracene, and cholanic acid (both 0.02 μg/μl in *n*-hexane, Sigma-Aldrich) was added prior to any Py-GC/MS measurement. After evaporation of the internal standards, 20 μl tetramethylammonium hydroxide (TMAH, 25% in methanol (MeOH), Sigma-Aldrich, Germany) was added for online derivatization of polyethylene terephthalate (PET) and polycarbonate (PC) to increase their detection sensitivity.

Data processing and polymer identification and quantification were performed using a combination of AMDIS (automated mass spectral deconvolution and identification system; National Institute of Standards and Technology, NIST) and Microsoft Excel 2013. Details of this new semi-automated identification and quantification approach presented here are given in ESM 2.pdf Paragraph S2, Tables S2–S5, and Fig. S3.

### Sample processing and data pretreatment for comparison

In this study, the use of a cover window was avoided and the particle numbers and sizes were determined using the AAP [23]. All given results were not blank corrected as the individual numbers and masses on the filters will be compared within this study. After data analysis, the determined polymer types were harmonized to allow a reasonable comparison between the results as presented in Table 1.

**Table 1:** Harmonized polymer types for the comparison between FTIR imaging datasets using the database of Primpke et al. [33] and the Py-GC/MS analysis described by Fischer and Scholz-Böttcher [56]

Harmonized polymer type	Py-GC/MS type	FTIR imaging types
PE	PE and copolymers	Polyethylene (PE), polyethylene oxidized, rubber type 3
PP	PP and copolymers	Polypropylene (PP)
PET	PET/PBT	Polyesters (PEST)
PS	PS and copolymers	Polystyrene (PS)
PVC	PVC, polyethylene chlorinated, polychloroprene	Polyvinyl chloride (PVC), polyethylene chlorinated, polychloroprene
PC	PC	Polycarbonate (PC)
PUR, PMMA	MDI-PUR, PMMA and all poly(alkyl methacrylate)s	Acrylates/polyurethanes (PUR)/varnish including polymethyl methacrylate (PMMA)
PA	PA6	Polyamide (PA)
Not assigned and excluded for comparison (polymers)		Cellulose chemical modified, nitrile rubber, polysulfone, polyether ether ketone, polylactic acid, polycaprolactone, ethylene-vinyl-acetate, polyimide, polyoxymethylene, polybutadiene, acrylonitrile-butadiene, rubber type 1, rubber type 2
Not assigned and excluded for comparison (minerals, coal, natural polymers)	–	Animal fur (natural polyamides), plant fibers (natural cellulose), quartz, chitin, charcoal, and coal

It is important to note that the polymer types derived via FTIR do not always contain solely one type of the material. In the case of PET and PA, Py-GC/MS is currently calibrated for PET and polybuthylethylene (PBT) as well as PA6, respectively, while the FTIR approach is designed to detect various types of polyester (PEST) and PA. The term PMMA for Py-GC/MS data includes at least shares of other poly(alkyl methacrylate)s if present in the samples [56] and all polymers included in this study that are present as parts of copolymers are included as their respective polymer share (cf. Table 1).

## Results and discussion

### Particle counts vs. masses in environmental samples

**Fig. 1:**
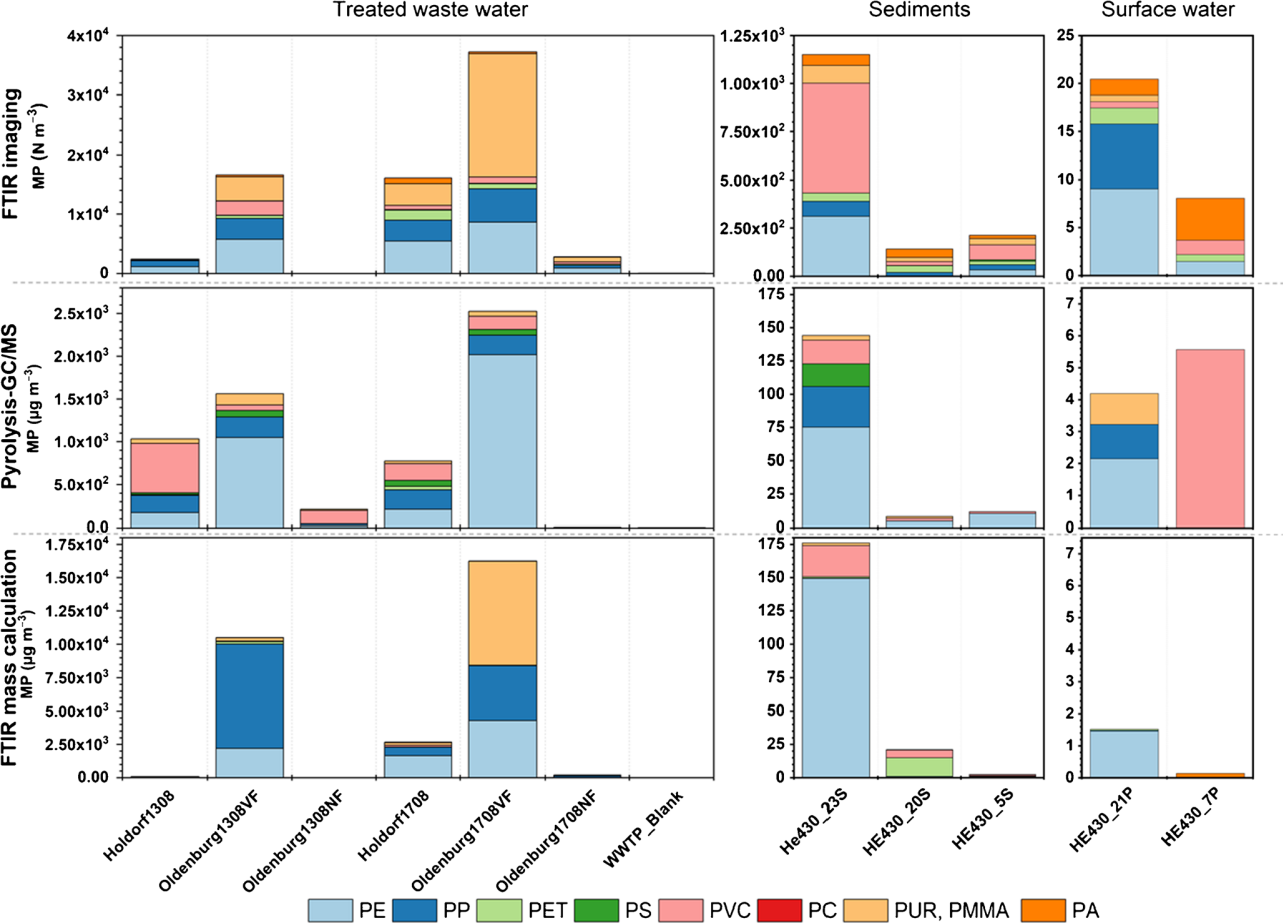
Quantitative MP composition data of individual polymers in three different environmental sample types based on determination by three different approaches. (1) Particle counts by FTIR imaging, (2) individual polymer masses directly determined by pyrolysis gas chromatography/mass spectrometry, and (3) individual polymer masses calculated from FTIR imaging particle numbers

The derived numbers and masses of the harmonized polymer types detected in individual samples are shown in Fig. 1 and in ESM 2.pdf Table S6. MP were identified in all investigated samples using Py-GC/MS and FTIR. The concentrations determined via Py-GC/MS ranged from 6 to 2525 μg m^−3^ for treated waste water, 4.2–5.5 μg m^−3^ for surface water samples, and 8–144 μg kg^−1^ for marine sediments. For these samples, the FTIR results range from 39 to 37223 N m^−3^ for treated waste water, 8–20 N m^−3^ marine water, and 143–1151 N kg^−1^ for marine sediments. Both methods found the same trends in MP contamination, with highest MP concentrations found at Oldenburg1708VF of the analyzed waste water samples and HE430_23S for sediments, respectively (see Fig. 1). These similar trends in particle and mass concentrations indicate a good overall comparability of the determined results. In the following, the results obtained by both analytical approaches will be discussed for the individual sample types. Additionally, mass calculations based on FTIR imaging data sets will be compared with those masses determined by Py-GC/MS.

### Treated waste water samples

The treated waste water (TWW) samples indicate a relatively high level of contamination (Fig. 1 left panel). FTIR imaging or Py-GC/MS analysis resulted in similar trends for five of seven samples. Two samples (Oldenburg1308NF and Oldenburg1708NF) showed differences either in absence or presence of MP. While the overall trend in MP abundances is similar, the polymer composition deviated. Here, FTIR indicated a particularly high presence of the PMMA/PUR group, and Py-GC/MS detected higher shares of PE and PVC. While the PP shares were comparable, the relative PET and PS contents varied between both methods. Conversion of FTIR into mass data resulted in an overall predominance of polyolefins and moreover for PMMA/PUR for Oldenburg1708VF. This mass calculation resulted in masses up to seven times higher (Oldenburg1708VF) compared with those determined via Py-GC/MS. Furthermore, the estimated masses reflect a high share of PP while PVC and PS were underrepresented or even missing on a relative scale. Regarding the procedural blank, only low numbers and small sizes (< 50 μm) of six different MP types were detected with FTIR (see ESM 2.pdf Table S6). Here, the mass of individual polymers was too small in most cases to be even detected by Py-GC/MS, and traces of PVC were quantifiable.

### Marine sediment samples

In sediments, intermediate MP contamination levels were found (see Fig. 1 middle panel). The general trend revealed in particle abundances of MP by FTIR imaging was reflected in MP mass concentrations analyzed via Py-GC/MS with the highest quantity at HE430_23S and the lowest at HE430_20S. The determined polymer composition is less variable for Py-GC/MS with a predominance of PE in all samples. PVC was detected in all samples, PMMA in two, while PP and PS are found in HE430_23S only. MP composition detected by FTIR showed the presence of PVC, PE, PMMA/PUR, PP, PES(T), and PA in all samples. In two of them (23S and 5S), PVC and PE are particularly abundant while PE is missing in HE430_20S.

Conversion of FTIR particles into masses led to a dominance of PE and PVC at least for the highest contaminated sediment (He430_23S) and He430_5S, while He430_20S contained PET and PVC. The overall MP mass range of the calculated and the measured data is approximately comparable, even though the calculated masses for HE430_5S are very low.

### Marine surface waters

At low MP contamination levels, high variations in relative abundances and polymer compositions are observed between the two analytical methods. Here, various polymer types are detected via FTIR imaging while Py-GC/MS is restricted to three or one cluster (Fig. 1, right column). In contrast, conversion of FTIR particle counts into masses reduces the polymer types to almost one prominent type, PE, and traces of others.

### Reasons for different relative abundances

#### Particle size

**Fig. 2:**
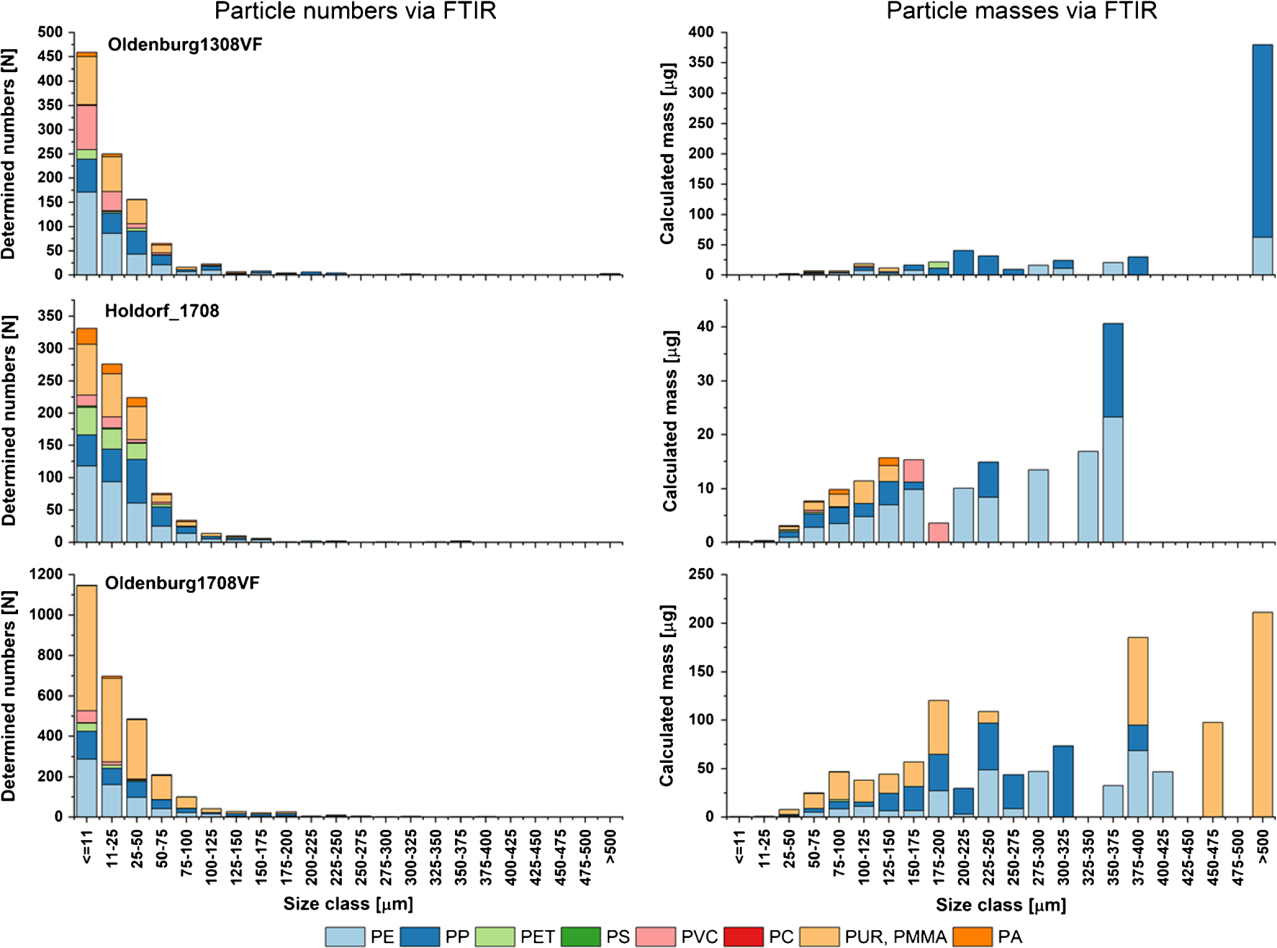
Particle numbers and estimated particle masses derived via FTIR imaging for selected samples of treated waste water using the harmonized polymer types

Measured particle abundances and mass trends for distinct polymers often differ, as expected, in the presented sample set. This is underlined by the calculated masses from FTIR imaging data. Here, abundance trends derived from the FTIR particle numbers often do not follow the calculated masses (see Fig. 1). At this point, the particle size and shape used for mass calculation becomes highly relevant. To highlight this general issue, the assigned FTIR polymer types in numbers and the resulting calculated masses are opposed as a function of their respective size class. For the TWW samples, only those containing larger numbers of particles > 200 μm are shown in Fig. 2. All other TWW samples are shown in ESM 2.pdf Fig. S4 and for detailed results of all samples ESM 3.tar.

In all cases, calculated masses were mainly driven by particles > 100 μm, which also led to the observed PE-PP-ratio inversion, e.g., for sample Oldenburg1308VF between particle and mass-related data. The high PP masses were caused mainly by a few large-sized PP particles, while the major part of PE particles is assigned to smaller sizes. The fact that masses complementarily determined by Py-GC/MS were at least one order of magnitude below the calculated ones indicates that the particle volume assumed of these PP particles led to an overestimation, which will be discussed later. Oldenburg1708VF also contains PUR/PMMA particles > 200 μm, which give correspondingly high masses.

For the samples Oldenburg1308VF and Holdorf1708, large particle counts but small particle sizes of PMMA/PUR and PVC were reflected in a low calculated mass equivalent.

**Fig. 3:**
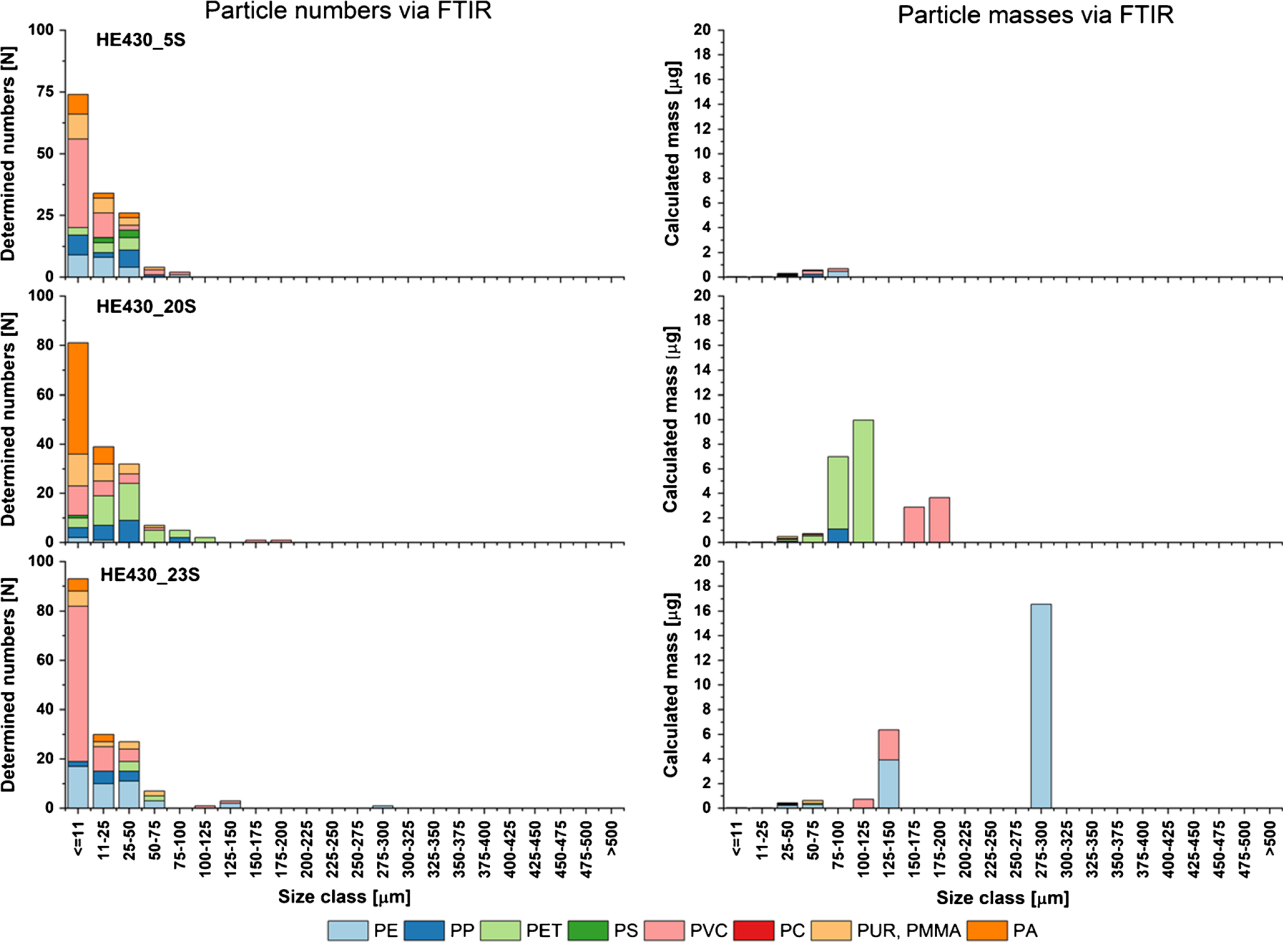
Particle numbers and calculated particle masses derived via FTIR imaging for the samples of marine sediments showing the harmonized polymer types

A similar trend was observed for the marine sediment samples (see Fig. 3). Again, mass calculations were mainly influenced by the presence of particles > 100 μm, here in particular from the polymer types PE, PET, and PVC. Interestingly, with lower overall particle abundances, the measured (Py-GC/MS) and calculated MP masses fell in the same mass range.

**Fig. 4:**
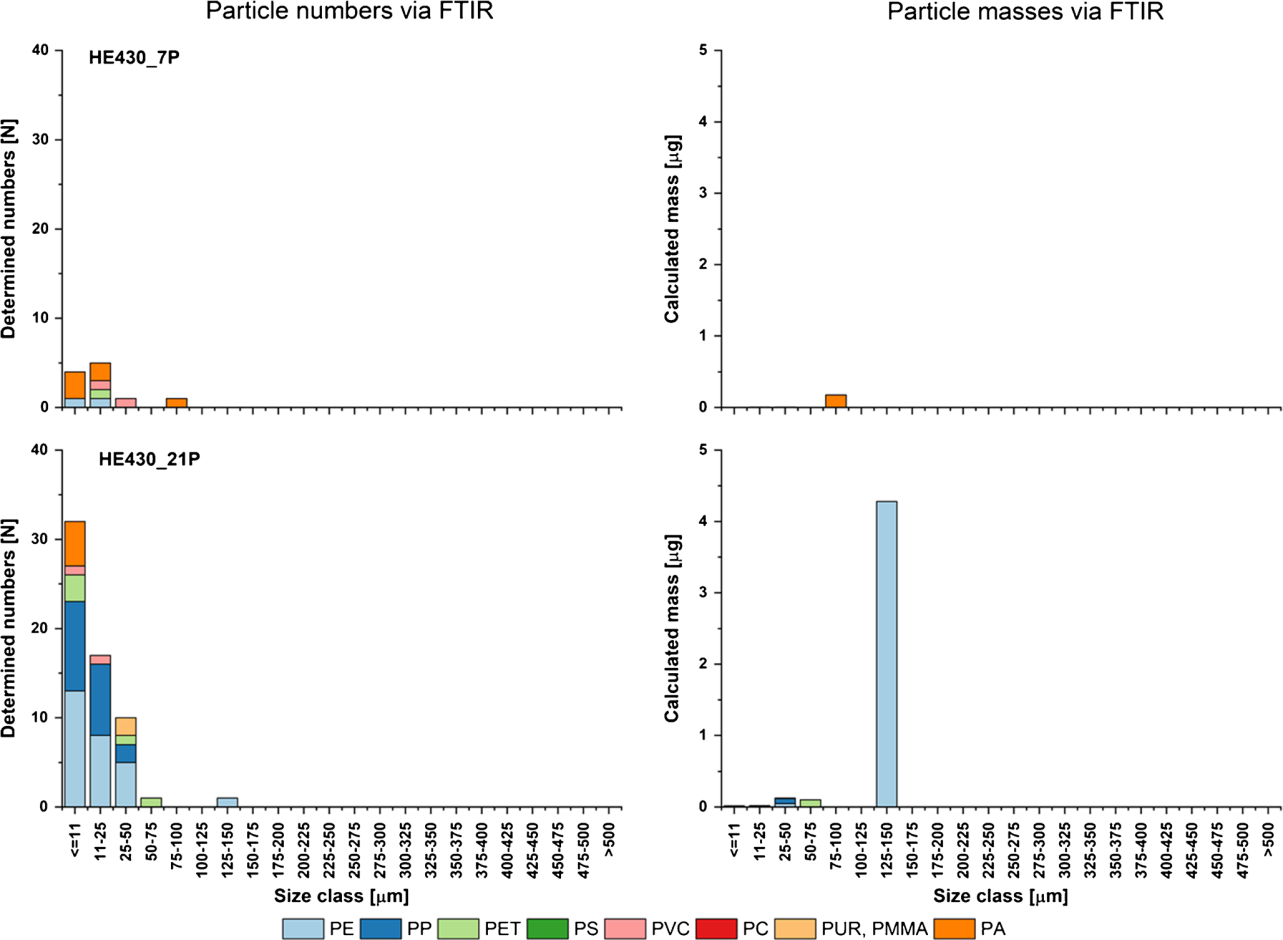
Particle numbers and estimated particle masses derived via FTIR imaging for the samples of marine surface waters showing the harmonized polymer types

For the marine water samples (see Fig. 4), the influence of particle size on mass estimation was even stronger. Again, the mass calculation changes the polymer composition remarkably compared with the FTIR particle abundance results. The diversity of polymer types for FTIR is mainly driven by particles with sizes < 50 μm, while the calculated masses are dominated by larger PE particles (Fig. 4) in a similar order of magnitude as the PE share determined by Py-GC/MS. In contrast, for HE430_7P, only very few particles of PE, PET, PVC, and PA < 50 μm, and one PA particle < 100 μm were detected. The latter represent the calculated mass exclusively. In contrast, Py-GC/MS measurement detected and quantified PVC only.

Figures 2, 3, and 4 point out an additional aspect regarding the FTIR analysis data and the qualitative polymer compositions resulting from these data, as the compositions can vary considerably in the respective size classes. Accordingly, the lower measurement limit of the instrument should not be underestimated when considering the overall relative polymer compositions based on particle counts.

#### Limit of quantification

Overall, most of the polymer diversity represented in the FTIR data is related to particles < 75 μm and their respective high abundances, but is almost lost after mass calculation due to their minor mass impact, as already pointed out in recent literature [46, 48]. Direct mass measurements by Py-GC/MS do not show this diversity which is due to targeted measurements further discussed later but also due to the limit of quantification (LOQ) (at the time the measurements were performed). Working with solid standards, the LOQ for Py-GC/MS was set by the available balance and ranges dependent on the polymer type between 0.7 and 1 μg absolute. Reduced to one single particle, this weight is roughly equivalent to a size between 50 and 200 μm. This has a large influence if the polymer compositions are investigated as the smaller particles have a stronger impact on the polymer composition for FTIR as it shows a high variability depending on the investigated size classes compared with Py-GC/MS. In contrast, the limit of detection (LOD equivalent to S/*N* > 3) is mostly far below 1 μg, again polymer-dependent and equivalent to much lesser particle sizes (cf. ESM 2.pdf Table S5). In ESM 2.pdf Table S7, an overview is given which polymers were quantified and detected via Py-GC/MS for the individual polymer types.

Too little masses are most plausibly the reason why PET, prominent in particle counts, does hardly appear on a mass scale. The exception (HE430_20S), where mass-relevant particles are present, might be traced back to the additional point that FTIR combines a larger number of PEST types in the database while Py-GC/MS just targeted PET in these measurements. The lack of PA detected in none of the samples by Py-GC/MS but frequently present in FTIR measurement and mass calculation (HE430_7P) might have a similar reason. While FTIR detected PA as a group, Py-GC/MS addressed only PA6. As both data sets can be reassessed in the future with extended polymer data/reference sets for better data harmonization, this finding cannot be finally valuated. The calculated PA amount in case of the sample HE430_7P falls below the LOQ for PA6 in the Py-GC/MS analysis, if this PA would be PA6. However, sample volume equivalent to the related polymer particle mass on the Anodisc filter is not sufficient to use the potential of Py-GC/MS for an informative MP composition in a reasonable way, here.

#### PVC, PS, and PP

Independent of sample origin, some differences in polymer composition were observed that have to be discussed on a more general level.

Disregarding the respective method, PVC was detected in almost all samples, but a systematic link between determined particle size and measured masses was, except for the sediment samples (see Figs. 1 and 3), often missing. PVC represent a consistent mass share in the Py-GC/MS measurement of the TWW samples (see Fig. 1), but even if the particle counts show the presence of PVC, these consistently small particles (<< 50 μm) have no impact on the mass calculation, and even few particles of 150–200 μm at sample Holdorf1708 did not count relative to the polyolefins. An outstanding example was Holdorf1308. Here, high masses of PVC were determined by Py-GC/MS but no PVC particle was detected via FTIR. The FTIR raw data of this particular sample (see ESM 1.xlsx) showed a high abundance of plant fibers over the full particle size range and elevated coal particles albeit < 75 μm were detected. Both might be potential precursors for benzene, the PVC indicator compound that is fairly weak regarding its polymer specificity. This potential interference needs further examination in this particular case, but can be almost excluded for the other TWW samples. This general discrepancy between FTIR and Py-GC/MS measurements needs further investigation in future studies.

Four of the analyzed samples (three WWTP and one sediment) show notably PS shares with Py-GC/MS detection, while they show low and small particle numbers in FTIR. As FTIR should be able to detect the related PS particles anyway, it is much more plausible that the PS detected by Py-GC/MS on the basis of its highly specific styrene trimer indicator product is derived from a PS copolymer, i.e., a styrene acrylate commonly used for paints and consumer products and possibly included in the PUR/PMMA/paint cluster of the FTIR data. An inconsistency pointing in the same direction was observed for sample Oldenburg1708NF where FTIR detected a highly mass-relevant PP particle while Py-GC/MS detected PP at trace levels only. This discrepancy was further investigated (see ESM 2.pdf paragraph S3, Fig. S5 and Fig. S6 for details) and the result indicates that this particle may either be a copolymer of PE and PP or a highly branched polymer with PE backbone. These particles as well as the PS masses stand exemplarily for actual limitations of the applied databases or method. The by now extensive FTIR reference database enables a critical re-investigation of the respective particle spectra. Even though Py-GC/MS data can be reinvestigated easily as well, the pyrograms and respective indicator ion(s) of the further suspected polymer types have to be known previously for a targeted search. This was not the case here as the number of polymers was restricted to nine representatives. Since the data were measured with an internal standard, a retrospective analysis might be performed at given times.

#### Other implications

In case of selected samples, a further plausible explanation for the observed differences could be given. The presence of fine red material (Oldenburg1308NF, see ESM 2.pdf Fig. S7a) or a fine opaque material (Holdorf1308, see ESM 2.pdf Fig. S7b), respectively, endured the applied sample treatment and hampered the FTIR measurements. This might have led to additional minor findings by FTIR by covering MP particles*.*

#### Target of the measurement

Finally, two key aspects have to be kept in mind when FTIR and Py-GC/MS polymer data are compared:

##### Polymer-type classification

As a result of highly developed spectral libraries and optimal particle separation out from each other, spectroscopically generated MP data represent often a broad suggestion of highly diverse polymer types that must be critically reviewed either manually or automatically. Accordingly, polymers are clustered to an acceptable extent to achieve an arguable set of polymers that enable further sample comparison. Clustering arguments base on spectral similarities in some cases includes different polymers in one cluster due to almost overlapping spectroscopic signals.

Even though extended pyrogram libraries exist for more than 1000 polymers and over 500 additives (F-Search, FrontierLab), they rely on single (particle) measurements. Py-GC/MS of environmental samples is a bulk measurement of the whole sample. The generated pyrograms sum up all generated indicator pyrolysis products disregarding their original precursor polymer. Ideally, any potential interference of natural organic materials should be excluded by preceded, adequate sample clean up. The resulting signal of a so-called polymer-specific indicator ion condenses all polymers or copolymers related to one respective polymer backbone. For quantification, this is finally expressed as the calibrated pure base polymer disregarding the original polymer type.

While FTIR detects the overall chemical absorption pattern directly related to functional groups inside the polymer after IR excitation, Py-GC/MS detects selected decomposition products of involved polymer chains as a result of pyrolysis. On macromolecular level, this can be of high importance for copolymers. Blends could be masked for FTIR by one compound with increasing mass ratio. Py-GC/MS detect decomposition products of both polymer types. This is consistent to the findings of Hermabessiere et al. [59] using Raman spectroscopy for one tested particle and Käppler et al. [60] for several particles and fibers using μATR-FTIR. Due to the presence of varnishes, it is most likely that these are not solely based on, e.g., acrylates, but may also contain crosslinking agents based on styrene or having chlororubber components. Both types are widely used as metal protection paints (styrene based) or for swimming pools and roofs (chlororubber). Similar results were also found by Hendrickson et al. [13] using ATR-FTIR on isolated particles for PE and PVC due to the chlorination of PE, which could not always be addressed by solely one technique.

##### LOD in relation to particle size or masses

In spectroscopy, the LOD is depending on the targeted size and instrumentation. The direct comparison of the determined polymer composition is therefore particle size-dependent as particle sizes typically follow a power law distribution. As already discussed, very small polymer particles are detected and quantified by Py-GC/MS measurements once they exceed a critical mass that defines the LOD and LOQ, respectively. Consequently, the contribution to mass increases with particle size. In addition, the mass is dependent on the shape of the particle (e.g., sphere versus fragment). The determination of this critical mass in relation to particle size and shape for the individual polymer types is one of the next challenges in the harmonization of FTIR and Py-GCMS methods with regard to MP analysis.

In consequence, both measurement principles discussed here have a different target and result in either particle numbers or masses. The quality of polymer detection is dependent on the kind of generated signal, its related quality, and the potentials of its interpretation.

Furthermore, our results indicate that the current mass calculation of Simon et al. [68] is currently limited if larger particles of complex shape are present and, thus, should be considered as an estimation.

#### Harmonization Py-GC/MS and mass calculation via FTIR imaging

For a discussion of relative overall composition patterns of a particular sample which is based on particle sizes, a clear hypothesis regarding the weighting of individual size fractions is needed.

While the MP trends of FTIR and Py-GC/MS are in good agreement overall, the derived mass calculations from FTIR data do not agree with the results of Py-GC/MS, as the masses determined were overestimated up to 6 times (Oldenburg1708VF) or underestimated by a factor of 10 (Holdorf1308) excluding the OldenburgNF samples due to the different targets and measurement principles mentioned above. Nevertheless, with decreasing contamination level, the accuracy of the estimation improved.

Still, at the current level, the polymer compositions are not comparable due to the different technical backgrounds. Here, the estimated mass concentrations are mainly underestimated; only for HE430_20S, a higher mass was estimated with different polymer composition compared with Py-GC/MS. Especially, a few larger particles caused severe differences as shown in sample Oldenburg1308VF, where large masses of PP were calculated but much lower when measured via Py-GC/MS. Based on Figs. 2, 3, and 4, it can be concluded that the accuracy of the estimate decreases with increasing particle size, as the underlying eclipse approach may overestimate the mass of the different particle shapes present. Here, the mass calculation is overestimating the particle volume and therefore the mass. Therefore, as suggested by Simon et al. [68], the results should be treated with care, since in particular large particles have a strong influence on the result.

**Fig. 5:**
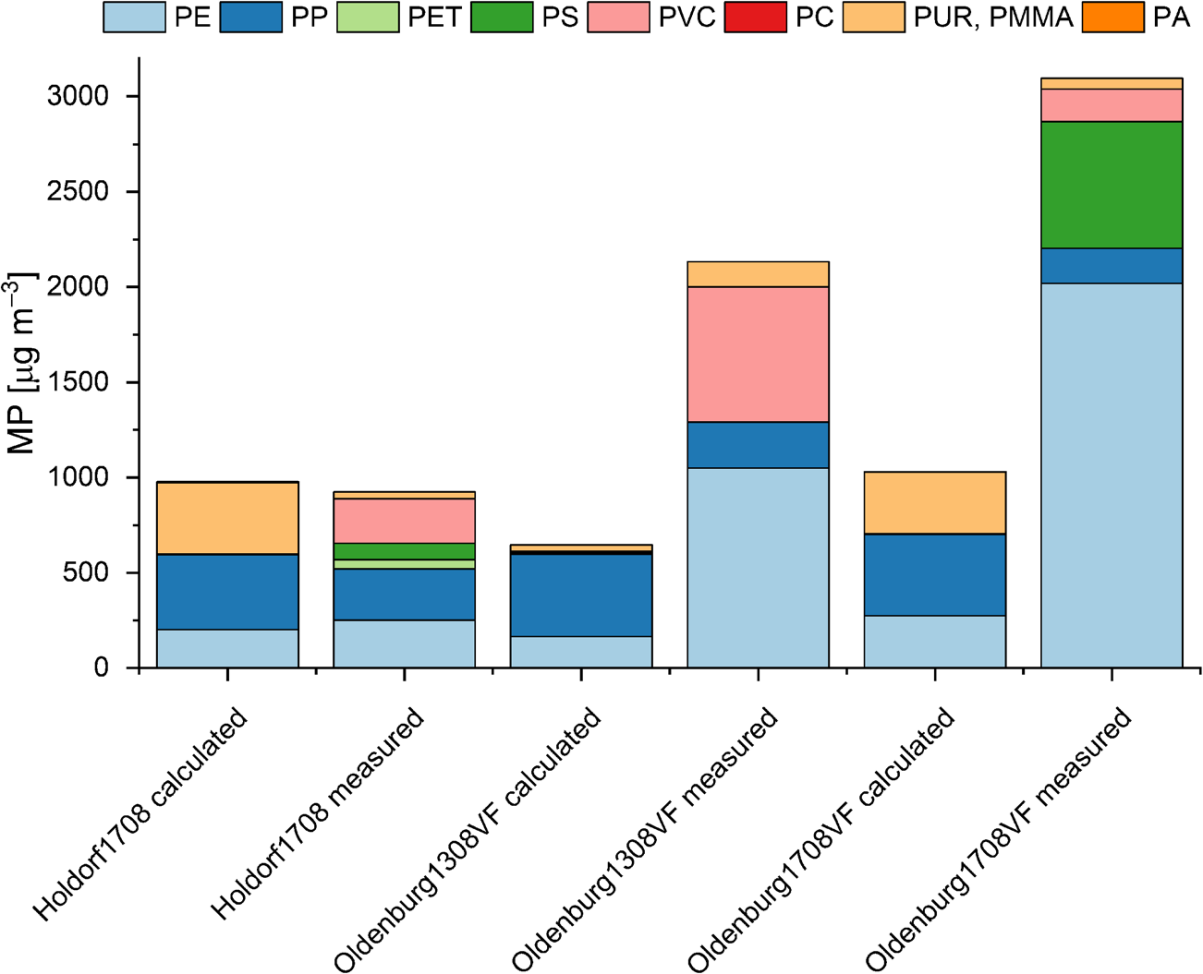
Alternatively calculated and measured (by pyrolysis gas chromatography/mass spectrometry) polymer mass for three chosen samples with a high number of particles > 100 μm

To overcome these limitations, a modified mass calculation was performed that combines various particle shapes and sizes present into a reference particle. For this purpose, the average particle length and diameter of each polymer type (see ESM 2.pdf Paragraph S4) was calculated and used as a reference particle in terms of mass. The area of the individual particles was divided by the reference area calculating the reference particles represented and multiplied by the reference particle mass. Compared with the mass calculation of Simon et al. [68], this weighted approach reduces the difference compared with the results of Py-GC/MS (see Fig. 5).

For the sample Holdorf1708, the calculated mass is similar to the one derived by Py-GC/MS while the masses for Oldenburg1308VF and Oldenburg1708VF are calculated lower. In all cases, the mass of PP is overestimated while the mass of PE is underestimated. Still, only a factor of 3 in difference is found indicating a better agreement possible using such a weight on the particle data.

Our results indicate that a calibration between Py-GC/MS and mass estimation may be possible, but this must be addressed by a specially designed investigation, which is currently hampered by the lack of suitable reference material and was therefore not part of this work.

## Conclusion

In total, the following table (see Table 2) supports and underlines the high complementary potential of both techniques. In this study, we carve out the complementarity of both techniques regarding identification as well as quantification of MP. Their respective use, separately or in combination, depends on the research/monitoring question asked. FTIR is the method of choice if any particle-related information is in the focus of interest. The number of detected polymer types is directly dependent on the respective polymer database used. Here, highly detailed information for specific particles can be preserved but have to be considered with experience as well. Py-GC/MS mass-related data reflect the respective specific polymer content more on a “bulk” level, were the number of targeted polymers (backbones) can be expanded continuously or even retrospectively. Retrospective quantification may be possible if internal standardization is used [57]. This compensates the destructiveness of the measuring principle. Thermoanalytical methods such as Py-GC/MS are so far the only possibility to determine reliable polymer-type masses. It has to be mentioned that thermal processes are complex and accompanying organic matrix products might cause interferences. Accordingly, appropriate caution and experience is necessary for data interpretation. Due to the fact that most risk assessment studies are linked to particle sizes, shapes, and numbers, spectroscopic techniques like FTIR imaging here are indispensable. For modelling and mass balance studies and their monitoring, Py-GC/MS is the method of choice while mass calculations based on FTIR particle counts need to be further investigated prior to future use to avoid a high risk of failure in the presence of many large particles of currently up to more than one order of magnitude. In an optimal workflow, a combination of both techniques should be used for identification and quantification [3, 75]. Here, ideally, the same samples are analyzed in a direct succession. As in this study, Anodisc filters are an ideal target. They enable first an analysis with FTIR imaging techniques for particle counts and can subsequently be directly transferred into pyrolysis cups for polymer mass determination via Py-GC/MS also suggested as ideal workflow for complementary MP quantification in literature [3].

**Table 2:** Comparison of FTIR imaging and Py-GC/MS for the analysis of microplastics in various environmental samples

	FTIR imaging	(Quantitative) Py-GC/MS
General information	General polymer type is identified as it is archived in the respective spectral library	Respective polymer backbones are determined based on targeted pyrolytic indicator products; different (co)polymers of same backbones are not distinguished
	Extend of detailed polymer information to be identified is directly related to the number of archived IR spectra	Number of identified basic polymers is restricted to those targeted but can be expanded by retrospective data analysis
	Datasets can be reanalyzed if new or better library are present	Datasets can be reanalyzed whenever indicator pyrolysis products for new polymers/clusters are defined if an internal standard was used for pyrolysis
	FTIR imaging contains not only particle data but also allows intra particle data analysis	Detailed chemical analysis is only possible on separated particles
Quantitation	Particle counts, related to size and particle shape of distinct polymers	Masses expressed as a basic polymer types that cover all polymers or the respective share of the respective polymer backbone
	Particle number increases with decreasing size; consequently, small sizes dominate counts and any resulting relative distribution pattern of polymers. These might vary seriously between different sample types	The masses directly represent the share of a respective polymer-(backbone). Relative polymer distribution patterns are mass-related and comparable between various samples in general
	Large particles are less pronounced into polymer composition	Determined masses are dominated by large particles
	Higher level of detail available for risk assessment (sizes and shapes)	MP mass loads enable a sample comparison on a trans ecosystematical, geospatial, as well as temporal scale. Any particle appearance (sizes and shapes) is neglected
	Value of particle-related data comparability increases with increasing relation of sample type and sampling region	Size class relation of data possible, requires prior size fractioning but raises analytical effort
	Conversion into masses is restricted to a rough mass estimation, limited at the current stage which needs to be further improved	Conversion to particle size classes possible via preceded filter cascades but of limited informative value due to non-perfect particle shape and size exclusion
Selected polymer level	Higher sensitivity for polymer groups like “PES,” “PAs,” “acrylates,” and PUR-based materials including varnish	High sensitivity for targeted polymers and higher sensitivity for PVC and PS
	Identification and quantification needs to overcome a distinct size threshold for reliable detection	Identification and quantification needs to overcome a distinct mass threshold for reliable detection
Additional aspects	Identification success can be hampered by the presence of inorganic materials	Identification is independent from inorganic matrix
	LOD needs to be reported and improved for further harmonization and comparison of polymer composition	LOD needs to be reported and improved for further harmonization and comparison of polymer composition
	Non-destructive: Analysis can be followed with other techniques	Destructive, but the use of internal standards allows the reanalysis of the derived data for new identifier ions data analysis

In general, the results of this study are of importance if source tracking of MP (secondary versus primary, manufacturer, etc.) is intended. Our findings at the current state-of-the-art implicate a complementary use of both techniques to achieve this goal. While FTIR detects a broad range and even very low numbers of smaller sized particles, Py-GC/MS, when exceeding a detection threshold, enables a condensed overview of polymer types represented by a shared chemical backbone expressed by basic polymer clusters (i.e., “PE” or “PS”). The data allow an insight on assigned or absorbed chemicals, additionally. The availability of information generated by both types of methods will enhance modelling and source tracking for future studies substantially.

## Electronic supplementary material

ESM 1 (XLSX 48.0 kb)

ESM 2 (PDF 2.37 mb)

ESM 3 (TAR 2.81  mb)

## Data Availability

All relevant data is provided via the Electronic Supplementary Material (ESM) via the files ESM 1.xlsx and ESM 3.tar. An executable file of python script is available online (https://drive.google.com/drive/folders/1O3vtsb963KoGwsTTGvDZgo8KUqjGjNAD?usp=sharing) and the measurement data is available upon reasonable request from the authors.
