# Supplementary material for: Comparison of pyrolysis gas chromatography/mass spectrometry and hyperspectral FTIR imaging spectroscopy for the analysis of microplastics
Source: Anal Bioanal Chem. 2020 Oct 26;412(30):8283–98. doi: 10.1007/s00216-020-02979-w (PMC7680748; doi:10.1007/s00216-020-02979-w)
Supplement: Supplementary file 2 — (PDF 2.37 mb) [file 216_2020_2979_MOESM2_ESM.pdf]

Analytical and Bioanalytical Chemistry

Electronic Supplementary Material

Comparison of pyrolysis gas chromatography/mass spectrometry and hyperspectral FTIR imaging spectroscopy for the analysis of microplastics

Sebastian Primpke, Marten Fischer, Claudia Lorenz, Gunnar Gerdts,
Barbara M. Scholz-Böttcher

Additional files available under 10.1007/s00216-020-02979-w

Paragraph S1: FTIR-Measurements and automated analysis

All FTIR imaging data were measured on a Hyperion 3000 µFTIR-Microscope coupled to a TENSOR II spectrometer (Bruker Optics GmbH, Ettlingen, Germany). The spectra were collected via a 64 x 64 focal plane array (FPA) detector with 4x4 binning (achieving a pixel resolution of 11.03 µm) on a merged area of 77 x 77 fields (13.6 x 13.6 mm) for surface water samples, 78 x 78 (13.8 x 13.8 mm) fields for sediment samples and 85 x 85 fields (15.2 x 15.2 mm) for treated waste water. In all cases the data was collected in the range of 3600-1250 cm^{-1} with a spectral resolution of 8 cm^{-1} and 6 co-added scans using Blackman-Harris Term 3 for apodization in accordance with literature[1]. A 4x lens was used to collect visual images of the sample surface, and 15x cassegrain objectives for IR measurements. All data was measured and collected by the Bruker OPUS 7.5 software (Bruker Optics GmbH, Ettlingen, Germany).

Analysis of the measured FTIR data was performed via the automated analysis pipeline first described in Primpke et al. [2]. To allow a harmonized analysis and comparison with the previous studies the previously published reference database [3] was used. This process ensures that all spectra were compared using vector normalized raw spectra and first derivatives spectra for the library search. Only if both spectra are matched with the same polymer type the spectra were marked as identified and further treated via image analysis. For this study the process was performed as a macro within the OPUS 7.2 software version, using HP KP719AV computers with an Intel© Core 2 Duo™ Processor, 8 GB RAM, AMD Radeon HD 5450 graphic card, an extra USB3.0 Controller card and a SANDISK Extreme 64 GB USB-Stick. The determined thresholds for QA/QC from the respective studies were applied for Image Analysis. Only for the cluster acrylates/polyurethanes and varnish a higher (1050) threshold needed to be applied for the WWTP samples. In addition, polycaprolactone was removed in accordance to the previous study[4] from these datasets.

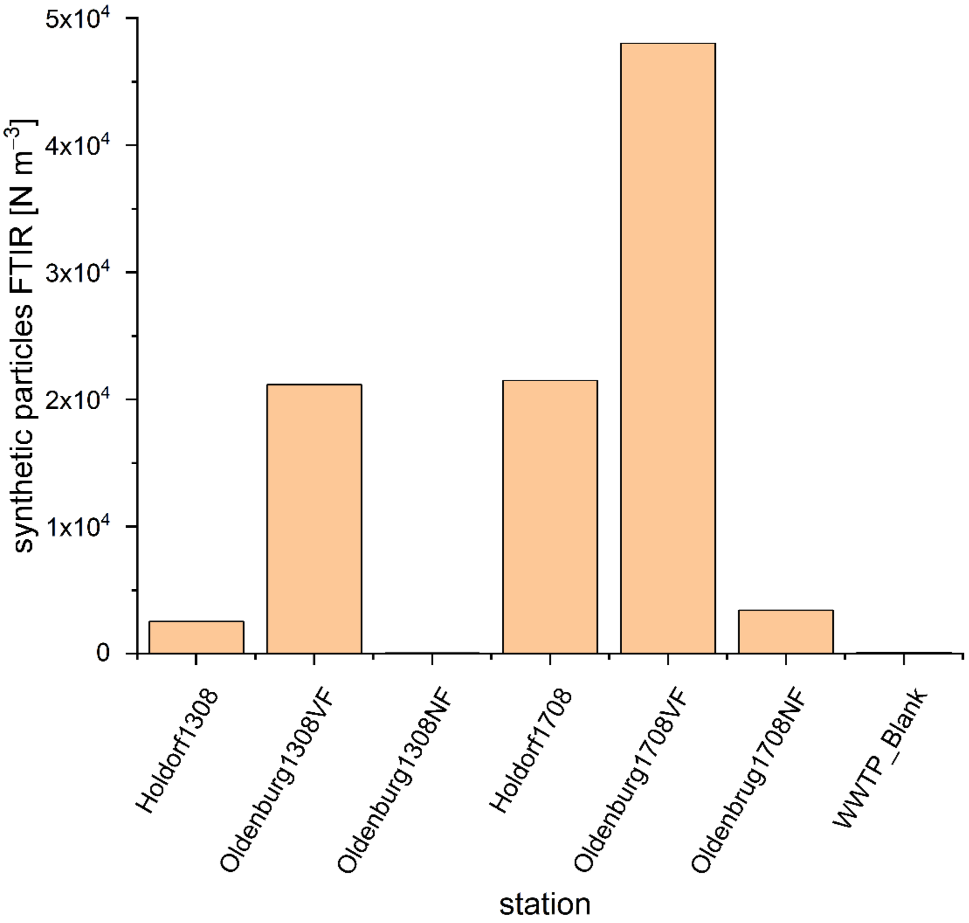

Fig. S1 Microplastic concentrations derived via FTIR imaging for all polymer types. See electronic supplementary materials ESM1.xslx for more details

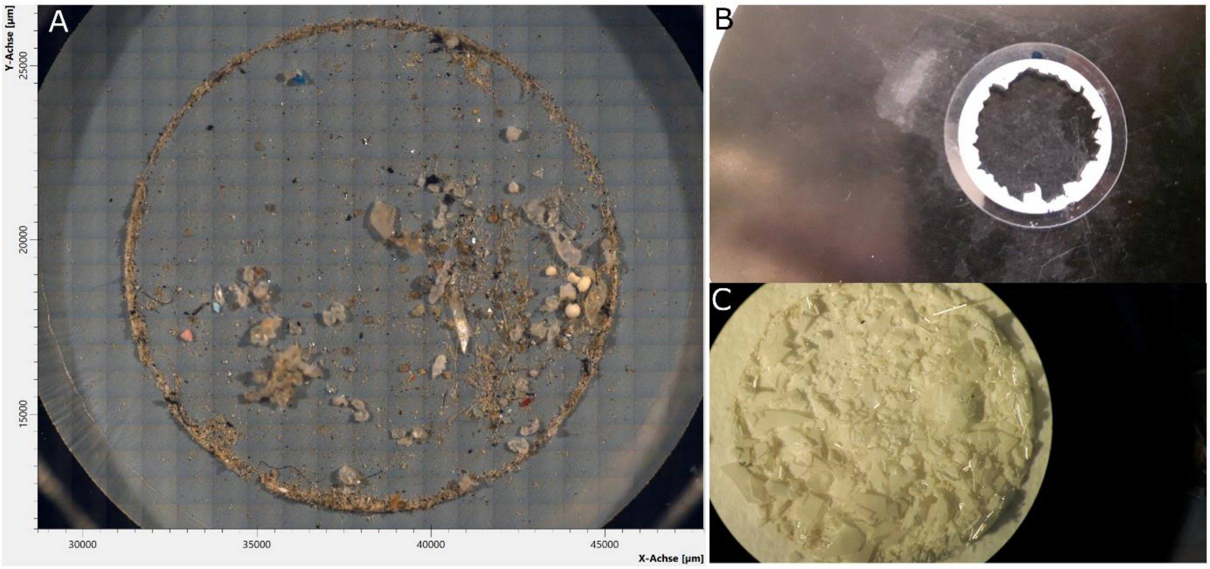

Fig. S2 Sample preparation for the Pyrolysis Gas chromatography/Mass spectrometry measurement: A) Sample on Anodisc during FTIR measurement; B) poked out Anodisc to remove the PP support ring; C) Transferred sample on glass fiber filter

Table S1 Pyrolysis parameters

**Micro furnace pyrolyzer**	**EGA/PY-3030D (FrontierLab)**
carrier gas	Helium
temperature	590°C
pyrolysis time	1 min
transfer line temperature	320°C
**Gas chromatograph**	**7890B (Agilent)**
injector	split/split less
mode	split 15:1
temperature	300°C
column	DB5 (J&W); 30 m x 0.25 mm ID, film thickness 0.25 µm
flow (const.)	0.8 ml/min
temperature program	35°C (2 min) → 310 °C (30 min) at 3°C/min
transfer-line temperature	280°C
**Mass spectrometer**	**MSD 5977A (Agilent)**
ionization energy	70 eV
source temperature	230°C
quadrupole temperature	150°C
scan rate	2.48 scans/s
scan range	50-650 amu

Paragraph S2: Py-GC/MS data processing, polymer identification and polymer quantification

Py-GC/MS data processing: The Py-GC/MS data was processed using the Automated Mass Spectral Deconvolution and Identification System (AMDIS; National Institute of Standards and Technology (NIST), USA). AMDIS performs a mass spectral deconvolution by grouping the ions that rise and fall at the same retention time and have the same peak shape. For detailed information on the deconvolution process see Stein [5]. The extracted (background free) mass spectra and the corresponding peak areas were then used for the further identification and quantification process.

Py-GC/MS - polymer identification: The samples were analysed for polyethylene (PE), polypropylene (PP), polystyrene (PS), polyethylene terephthalate (PET), polyvinylchloride (PVC), polymethyl methacrylate (PMMA), polycarbonate (PC), polyamide 6 (PA6) and methylendiphenylisocyanate-polyurethane (MDI-PUR). Polymer identification was performed with polymer-specific indicator compounds (listed in the supplement in Table S2) according to Fischer & Scholz-Böttcher [6] and extended by Fischer & Scholz-Böttcher [7]. The data evaluation was carried out with AMDIS 2.72 (NIST, USA). The mass spectra of the respective indicator compounds and their corresponding retention indices (RI) (according to van den Dool and Kratz [8]) were extracted from polymer standards (PE, PP, PS, PET, PVC, PMMA, PC, PA6 and MDI-PUR) and included in a custom made AMDIS target library.

Identification of indicator compounds within the chromatograms of the samples was performed as a routine of AMDIS by comparing the extracted mass spectra with the mass spectra from the target library. The similarity of the spectra (presence/absence of ions and their intensities relative to the base peak) is expressed with a match factor, where a match factor of 100 corresponds to identical spectra. For a positive identification of a target compound, a minimum match factor of 80 had to be fulfilled.

In each measurement sequence the RI was recalibrated using a PE standard from the sequence. The retention times of the *n*-alkanes (*n*-C_{7-31}-alkanes) are assigned to their respective RI [8] (e.g. retention time of *n*-C_{15} = RI 1500, retention time of *n*-C_{16} = RI 1600). To minimize false-positive results, the RI of the positively identified indicator compound was checked against the RI from the target library. The RI window was set to the RI of the respective target compound ± 40 (example: *n*-C_{15} alkane - RI: 1500, RI window: 1460 - 1540). Any suspected compound outside this RI window is generally excluded. The identified target compounds were frequently checked after automatic identification by personal inspection. Target compounds with a slightly lower matching factor >70 (50 for PE indicator compounds) were also checked. This was particularly necessary for the PE indicator compounds (*n*-

alkadienes), whose mass spectra often show a lower matching factor - especially if they are only present in traces.

Py-GC/MS - polymer quantification: Polymer quantification was performed for PE, PP, PS, PET, PVC, PMMA, PC, PA6 and MDI-PUR. For calibration standards were weight from 0.5 to 50 µg directly into the pyrolysis cups using a Cubis Ultramicro balance MSE2.7S-000-DM (Sartorius, Germany; readability 0.0001 mg, repeatability 0.00025 mg). Standards used for calibration are listed in the supplement (supplement Table S3). The internal standardization of the pyrolysis process was performed after Fischer & Scholz-Böttcher [7] and 50 µl of a mixture of 9-Tetradecyl-1,2,3,4,5,6,7,8-Octahydro anthracene (0.01 µg/µl in *n*-hexane, Sigma/Aldrich) 9-Dodecyl-1,2,3,4,5,6,7,8-Octahydro anthracene and cholanic acid (both 0.02 µg/µl in *n*-hexane, Sigma/Aldrich) were added to the standards and samples prior to Py-GC/MS measurements. After evaporation for 10 min at room temperature 20 µl of TMAH (25% in MeOH) were added for thermochemolysis. The solvent (MeOH) was evaporated (RT, 30 min).

Individual calibration curves were created using Microsoft Excel 2013. For this purpose, the ratios of preselected indicator compounds to internal pyrolysis process standards (ISTDpy) were used (using the areas of the deconvoluted peaks). For this internal calibration of the pyrolysis process the matching ISTDpy (c.f. Fischer & Scholz-Böttcher [7]) was selected for each polymer individually, based on the highest coefficient of determination (r^2 - as a measure of the quality of the linear fit) and the lowest process standard deviations (s_{x0} - as a measure of the error), calculated from the calibration data (see Table S4). All calibration curves followed a linear regression. Representative calibration curves for all polymers are given in Fig. S3. The bands of confidence and prediction at 95% confidence level and r^2 were calculated with Origin 2018G (OriginLab corporation), s_{x0} was calculated after DIN 38402 A51.

LODs and limits of quantification (LOQ) of this method are discussed in Fischer & Scholz-Böttcher [7]. The signal-to-noise (*S/N*) ratios of the lowest calibration standards are given in the supplement (see Table S5).

The samples were measured in different measurement campaigns. Each sequence contained 2 to 5 samples and, blanks as well as 4 to 8 individual standard mixtures to perform individual calibrations for every polymer and sequence (see Table S4).

Table S2 Polymer specific indicator compounds under conditions of thermochemolysis

Polymer	Abbre-viation	Characteristic decomposition product(s)	RI [a]	M (*m/z*)	Indicator ions (*m/z*)
Polyethylene	PE	Alkanes (e.g. C_{20})	2000	282	85
		α-Alkenes (e.g. C_{20})	1994	280	**83**
		**α,ω-Alkenes (**e.g. C_{20}**)**	1987	278	**82**
Polypropylene	PP	**2,4-Dimethylhept-1-ene**	832	126	126, **70**
		2,4,6,8-Tetramethyl-1-undecenes[b]	1306	210	100, 69
		2,4,6,8-Tetramethyl-1-undecenes[c]	1315	210	100, 69
		2,4,6,8-Tetramethyl-1-undecenes[d]	1323	210	100, 69
Polystyrene	PS	Styrene	890	104	104
		2,4-Diphenyl-1-butene	1720	208	91
		**2,4,6-Triphenyl-1-hexene**	2440	312	**91**
Polyvinyl chloride	PVC	**Benzene**	738	78	**78**
		Chlorobenzene	840	112	112
Poly(methyl methacrylate)	PMMA	Methylacrylate	726	86	55
		**Methyl methacrylate**	775	100	**100**, 69
Polyamide	PA6	**ε-Caprolactam**	1257	113	**113**
		***N*-methyl caprolactam**[e]	1224	127	**127**
Polyethylene terephthalate	PET	**Dimethyl terephthalate**[e]	1504	194	**163**
Polycarbonate	PC	*p*-Methoxy-tert-butylbenzene[e]	1240	242	164, 149
		**2,2-Bis(4'-methoxy-phenyl)propane**[e]	2065	256	256**, 241**
MDI-Polyurethane	MDI-PUR	4,4'-Methylenbis(*N*-methylaniline) [e]	2330	226	226
		N,N-Dimethyl-4-(4-methylamino)benzylanilin[e]	2341	240	240
		**4,4'-Methylenbis(*N,N*-dimethylaniline)** [e]	2354	254	253, **254**

[a]RI = Retention index calculated after Van Den Dool 1963, DB-5 column; M = molecular ion, m/z = mass to charge ratio; [b]Isotactic. [c]Heterotactic. [d]Syndiotactic. [e]Only after TMAH teatment; bold: indicator ions used for calibration

Table S3 List of standards used for identification/quantification

Polymer standard	Abbre-viation	Further specifications	Use (examples)	Source of supply
Polyamide 6 (K891), Akulon® K222-D	PA6	Low viscosity	Consumer durables, convoluted tubes	Ter Hell GmbH, Hamburg, Germany
Polycarbonate, Makrolon 2558	PC	SM (solvent method) PC	Households products/ Consumer Goods (Toys),	Bayer Material Science
Polyethylene, Lupolen 4261 AG UV	HDPE	High density	injection molding	LyondellBasell
Polyethylentereph-thalate, NEOPET 80	PET			Neogroup
Polymethylmeth-acrylate, PLEXIGLAS® 7N	PMMA	Thermoplast	Optical waveguides	Plexiglas
Polypropylene, HL508FB	PP	Homopolymer, isotactic	for medical devices, secondary packaging, infusion bags	Borealis
Polystyrene, TOTAL PS impact 7240	PS	High impact PS for extrusion industry	Dairy sheets, dairy pots	Ter Hell GmbH, Hamburg, Germany
Polystyrene, Styrolution PS 158N/L	PS	Raw material	Packaging material, foams	IINEOS Styrosolution
Polyurethane	PUR	MDI - Thermoplast		GEBA GmbH
Polyvinylchloride, Vinnolit S 3268	PVC	Hard PVC, raw material	Extruding mass	Vinnolit

*The additive content of the used standards is about 1-2% as it is reported for such common thermoplastic materials and were ignored regarding calibration. Effects of standards from different suppliers as well as comparable consumer plastics (here PE, PP, PVC, the latter of known additive content) were tested and discussed in Fischer & Scholz-Böttcher 2017 and reasoned to be neglectable.

Table S4 Overview of Py-GC/MS calibration parameters

	PE	PP	PET	PS	PVC	PC	PMMA	PA6	PUR
**Sequence 180612**									
Inj. Std.	DOHA	DOHA	Cholan.	DOHA	DOHA	DOHA	DOHA	DOHA	DOHA
b	-4.21E-02	-4.73E-01	-5.74E-01	-3.53E-02	-6.05E-01	-1.72E-01	-1.15E+00	-1.31E+00	-5.07E-01
slope	1.36E-02	1.58E-01	3.23E-01	4.51E-02	1.49E-01	8.25E-01	5.10E-01	2.48E-01	5.76E-02
r^2	0.97	0.96	0.97	0.97	0.94	0.98	0.93	0.97	0.97
s_{x0}	3.3	4.0	1.3	2.1	5.3	2.2	2.5	3.5	3.2
n	6	6	6	4	5	6	6	5	4
**Sequence 180530**									
	PE	PP	PET	PS	PVC	PC	PMMA	PA6	PUR
Inj. Std.	DOHA	DOHA	DOHA	DOHA	DOHA	DOHA	DOHA	DOHA	DOHA
b	-3.79E-03	6.12E-02	-3.45E-01	4.49E-02	1.28E-02	4.50E-01	2.02E-01	-7.58E-02	-4.76E-01
slope	6.76E-03	6.47E-02	2.56E-01	7.18E-02	1.82E-02	1.06E+00	1.28E-01	1.06E-01	4.72E-02
r^2	1.00	1.00	1.00	0.98	1.00	0.98	0.98	0.99	0.99
s_{x0}	1.1	0.9	0.4	1.2	0.9	0.4	0.7	1.0	2.6
n	6	6	5	5	5	4	5	4	4
**Sequence 180509**									
	PE	PP	PET	PS	PVC	PC	PMMA	PA6	PUR
Inj. Std.	DOHA	DOHA	DOHA	DOHA	DOHA	DOHA	DOHA	DOHA	DOHA
b	-1.67E-02	2.28E-02	-5.33E-03	2.31E-02	-9.31E-04	6.64E-01	4.67E-02	-1.54E-01	-1.70E-01
slope	7.49E-03	3.25E-02	1.06E-01	7.09E-02	7.34E-03	1.79E-01	8.50E-02	1.00E-01	3.69E-02
r^2	0.97	1.00	0.99	0.89	1.00	0.94	0.94	0.97	1.00
s_{x0}	3.5	1.5	0.7	2.5	1.5	1.8	2.7	3.8	0.4
n	5	5	4	4	4	5	4	4	4
**Sequence 181016**									
	PE	PP	PET	PS	PVC	PC	PMMA	PA6	PUR
Inj. Std.	Cholan.	Cholan.	Cholan.	Cholan.	DOHA	DOHA	DOHA	Cholan.	DOHA
b	-6.80E-03	3.25E-02	-1.75E-01	-2.66E-01	1.02E-02	2.99E-01	-1.72E-02	-3.13E-01	-8.56E-01
slope	6.34E-03	4.56E-02	1.90E-01	1.73E-01	8.59E-03	4.95E-01	3.95E-02	7.75E-02	8.49E-02
r^2	1.00	0.98	0.99	0.96	0.98	0.96	0.99	0.99	1.00
s_{x0}	1.046	2.004	0.707	1.598	2.213	1.2	0.655	2.086	1.039
n	8	8	8	6	8	7	6	5	4
**Sequence 190115**									
	PE	PP	PET	PS	PVC	PC	PMMA	PA6	PUR
Inj. Std.	Cholan.	Cholan.	Cholan.	Cholan.	Cholan.	Cholan.	Cholan.	Cholan.	Cholan.
b	8.55E-04	2.06E-03	2.18E-01	-3.30E-02	-1.10E-02	-2.96E-01	2.54E-02	8.76E-04	-5.40E-02
slope	3.17E-03	4.30E-02	1.78E-01	7.11E-02	5.78E-03	4.75E-01	6.52E-02	2.61E-02	1.01E-02
r^2	1.00	0.96	0.96	1.00	0.98	0.99	0.91	0.97	0.96
s_{x0}	0.1	4.2	2.6	0.7	2.5	0.5	2.7	4.0	4.6
n	4	5	6	4	5	4	5	4	4

Table S5 Signal-to-noise ratios of lowest Py-GC/MS calibration standards

Polymer	Indicator compound	Indicator ion *m/z*	low. calibration point µg	*S/N*
PE	*n*-C16-26-alkadienes	82	0.7	>21
PP	2,4-Dimethylhept-1-ene	70	0.8	148
PET	dimethyl terephthalate	163	0.7	114
PS*	2,4,6-Triphenyl-1-hexene	91	0.9	208
PVC	benzene	78	0.8	110
PC	dimetyhl bisphenol-A	241	0.9	819
PMMA	methyl methacrylate	100	0.8	153
PA6	ε-caprolactame + *N*-Methyl-Caprolactame	113+127	1	77
PUR	4,4'-Methylenbis(*N,N*-dimethylaniline)	254	0.9	78

- LOD 0.03 µg (cf. Fischer et al. 2019)

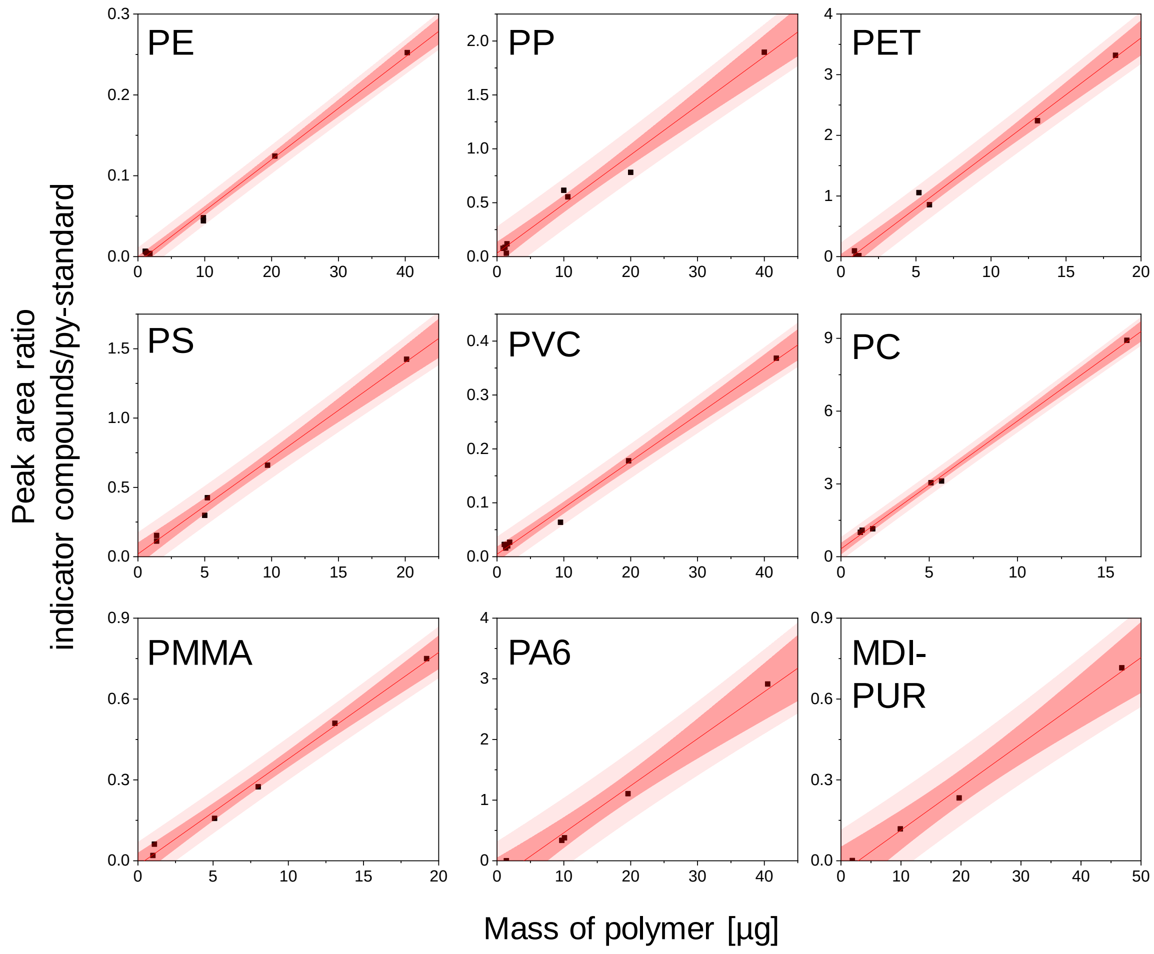

Fig. S3 Example calibration curves (sequence 181016)

Table S6 Harmonized analysis results

Sample Name	Measurement	unit	PE	PP	PET	PS	PVC	PC	PMMA/PUR	PA
Holdorf1308	FTIR	N m^{-3}	1152	1015	98	0	78	0	78	0
Oldenburg1308VF	FTIR	N m^{-3}	5742	3531	512	33	2393	33	4059	314
Oldenburg1308NF	FTIR	N m^{-3}	0	35	0	0	4	0	0	0
Holdorf1708	FTIR	N m^{-3}	5064	3650	1200	82	986	0	8483	871
Oldenburg1708VF	FTIR	N m^{-3}	8632	5657	867	53	1067	0	20653	294
Oldenburg1708NF	FTIR	N m^{-3}	946	451	207	10	338	17	792	80
Blank	FTIR	N m^{-3}	15	4	29	0	2	0	2	20
HE430_P21	FTIR	N m^{-3}	9	7	2	0	1	0	1	2
HE430_P7	FTIR	N m^{-3}	1	0	1	0	1	0	0	4
HE430_S23	FTIR	N kg^{-1}	313	78	43	0	569	0	92	57
HE430_S20	FTIR	N kg^{-1}	3	18	35	1	21	0	21	44
HE430_S5	FTIR	N kg^{-1}	34	27	18	8	78	0	31	18
Holdorf1308	Mass calculation	µg m^{-3}	70	24	0	0	0	0	0	0
Oldenburg1308VF	Mass calculation	µg m^{-3}	2215	7800	172	0	48	0	254	4
Oldenburg1308NF	Mass calculation	µg m^{-3}	0	1	0	0	0	0	0	0
Holdorf1708	Mass calculation	µg m^{-3}	1907	986	14	0	257	0	740	47
Oldenburg1708VF	Mass calculation	µg m^{-3}	4296	4105	29	1	2	0	7799	14
Oldenburg1708NF	Mass calculation	µg m^{-3}	17	121	6	0	7	0	51	0
Blank	Mass calculation	µg m^{-3}	0	0	0	0	0	0	0	0
HE430_P21	Mass calculation	µg m^{-3}	1	0	0	0	0	0	0	0
HE430_P7	Mass calculation	µg m^{-3}	0	0	0	0	0	0	0	0
HE430_S23	Mass calculation	µg kg^{-1}	150	0	1	0	23	0	2	0
HE430_S20	Mass calculation	µg kg^{-1}	0	1	14	0	6	0	0	0
HE430_S5	Mass calculation	µg kg^{-1}	1	0	0	0	1	0	0	0
Holdorf1308	Py-GC/MS	µg m^{-3}	174	199	10	21	576	0	53	0
Oldenburg1308VF	Py-GC/MS	µg m^{-3}	1049	241	0	74	66	0	132	0
Oldenburg1308NF	Py-GC/MS	µg m^{-3}	26	17	0	0	156	0	14	0
Holdorf1708	Py-GC/MS	µg m^{-3}	212	227	41	72	192	0	31	0
Oldenburg1708VF	Py-GC/MS	µg m^{-3}	2018	229	0	67	154	0	57	0
Oldenburg1708NF	Py-GC/MS	µg m^{-3}	0	0	0	0	11	0	0	0
Blank	Py-GC/MS	µg m^{-3}	0	0	0	0	6	0	0	0
HE430_P21	Py-GC/MS	µg m^{-3}	2	1	0	0	0	0	1	0
HE430_P7	Py-GC/MS	µg m^{-3}	0	0	0	0	6	0	0	0
HE430_S23	Py-GC/MS	µg kg^{-1}	75	31	0	17	18	0	4	0
HE430_S20	Py-GC/MS	µg kg^{-1}	5	0	0	0	2	0	1	0
HE430_S5	Py-GC/MS	µg kg^{-1}	10	0	0	0	1	0	0	0

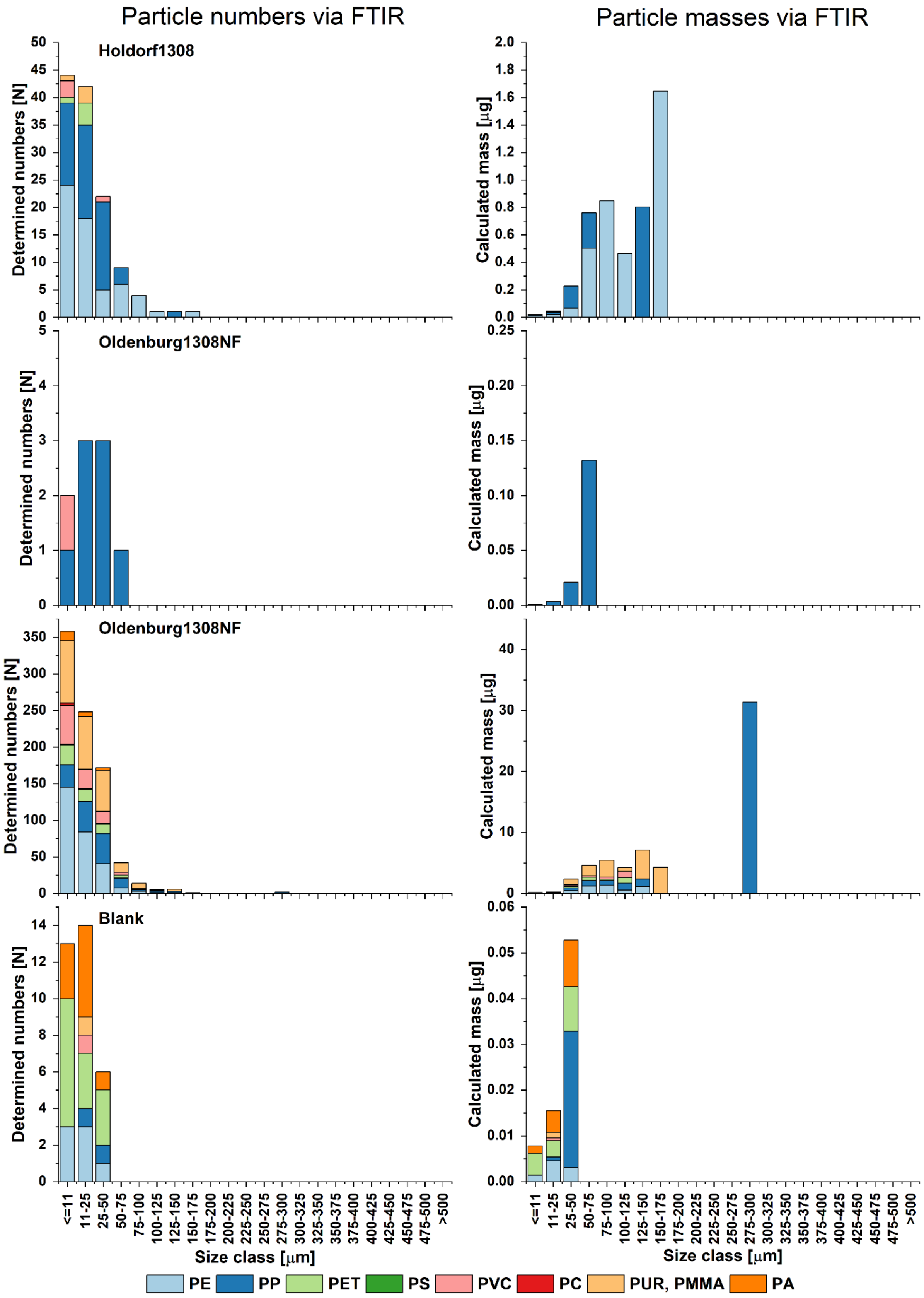

Fig. S4 Particle numbers and estimated particle masses derived via FTIR imaging for the residual samples of treated waste water using the harmonized polymer types

Table S7 Overview on the quantified (Q) and detected (D) polymers using Py-GC/MS in the investigated samples. PE: polyethylene; PP: polypropylene, PET: polyethylene terephthalate, PS: polystyrene, PVC: polyvinylchloride, PC: polycarbonate, PMMA: polymethyl methacrylate, PA6: polyamide Nylon 6, PUR: polyurethane

	PE	PP	PET	PS	PVC	PC	PMMA	PA6	PUR
Holdorf1308	Q	Q	Q	Q	Q	**D**	Q		
Oldenburg1308VF	Q	Q		Q	Q		Q		
Oldenburg1308NF	Q	Q			Q		Q		
Holdorf1708	Q	Q	Q	Q	Q	**D**	Q		
Oldenburg1708VF	Q	Q		Q	Q	**D**	Q		
Oldenburg1708NF		**D**			Q		**D**		
Blank_TWW					Q				
HE430_P21	Q	Q					Q		
HE430_P7					Q				
HE430_S23	Q	Q		Q	Q		Q		
HE430_S20	Q				Q		Q		
HE430_S5	Q				Q				

Paragraph S3: To exclude a swap of samples during sample transfer, pictures of the Anodisc filter from FTIR measurement and GF filter for Py-GC/MS analyses were compared. In both cases a prominent particle (labeled with a square in Fig. S5) identified as PP by FTIR was present, which ruled out the possibility of confusion. Accordingly, we reassessed the FTIR data of this particle (see Fig. S6). While the FTIR spectrum fits to PP due to the signal set in the wavenumber region of 1500 cm^{-1} to 1250 cm^{-1} the peak at 1400 to 1325cm^{-1} has an untypical lower intensity compared to the PP reference spectrum and displays shoulders (see Fig. S6a). Further, a weak peak typically for PE between 2685 cm^{-1} to 2575 cm^{-1} is present together (see Fig. S6c) with a peak typical for PP at 2750 cm^{-1} to 2700 cm^{-1}. These signals are indicating that the particle is either a copolymer of PE and PP or a highly branched polymer with a PE backbone, which was assigned to the PP polymer type. This assumption is supported by the integration of the peak regions of 1500 – 1400 cm^{-1} and 1400 – 1325 cm^{-1} (see Fig. S6 d and e) which indicate a changing ratio on the left lower side of the particle. A similar result was found if the absolute peak intensity was investigated (see Fig. S6 f and g).

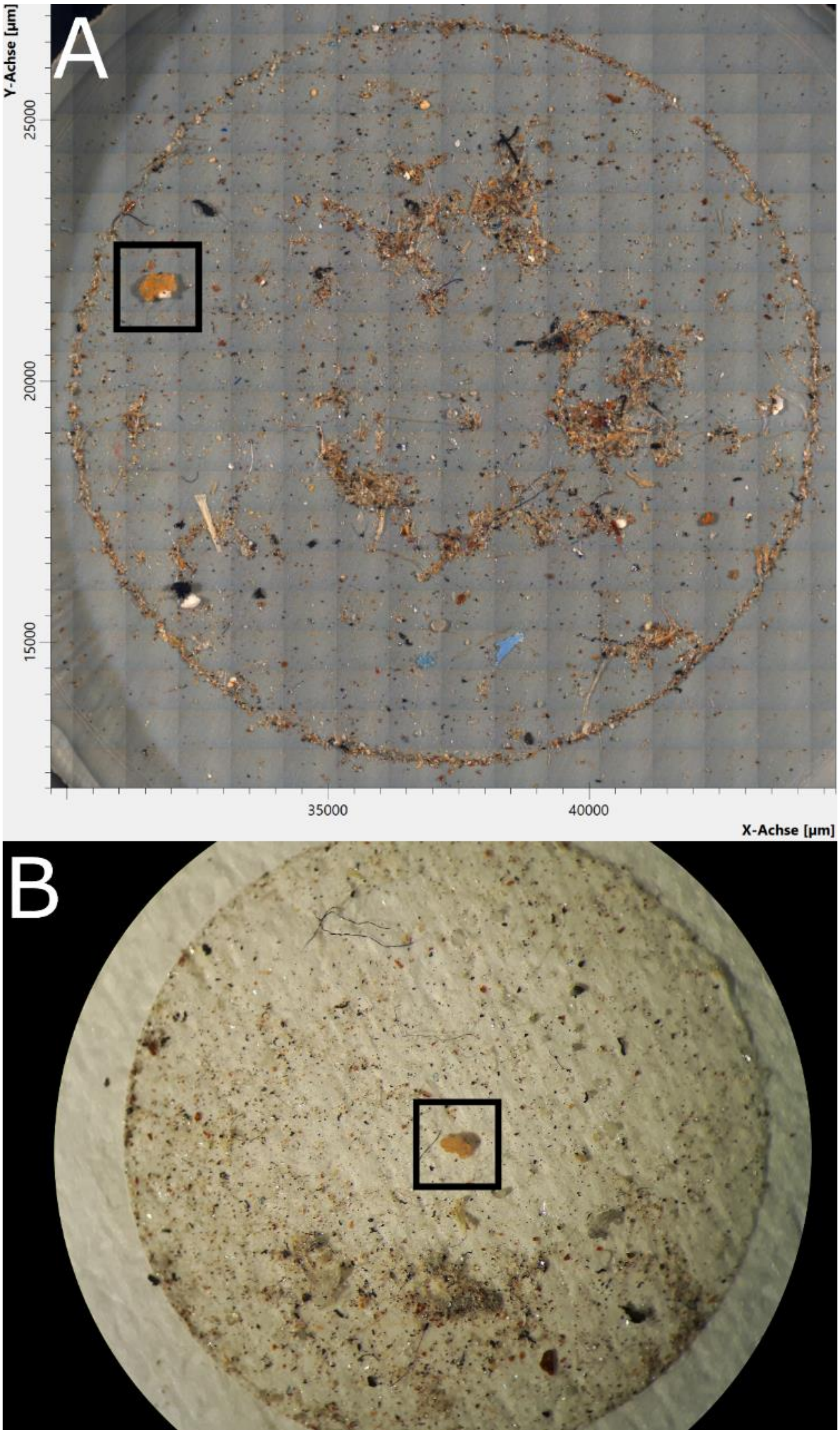

Fig. S5 Sample Oldenburg1708NF prior to analysis via FTIR (A) and pyrolysis GC/MS (B) with the orange PP particle visible in both pictures highlighted by black squares

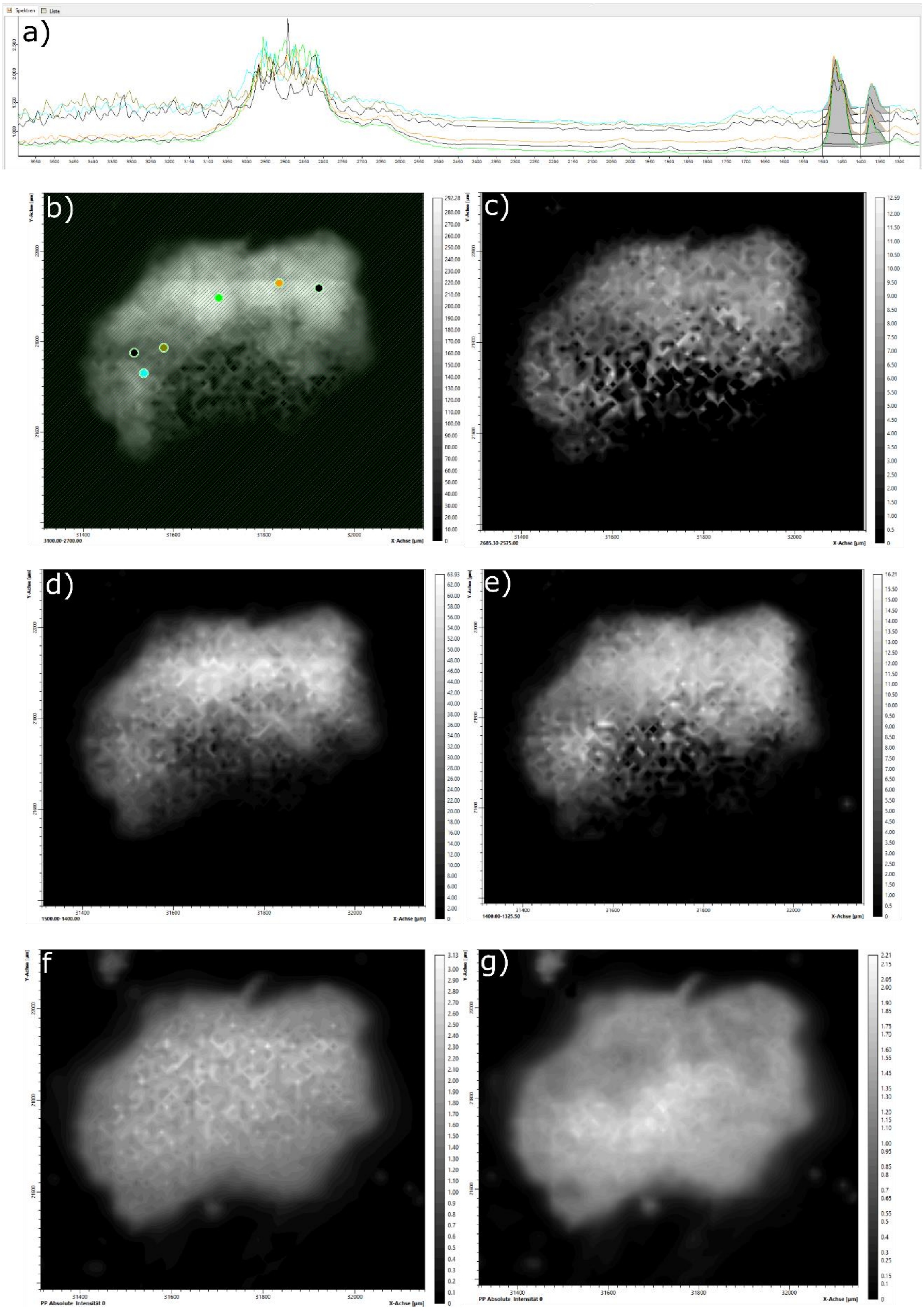

Fig. S6 False color images of the selected particle in the black square in Fig. S5. a) Selected spectra of the particle; b) integrated region of 3100 – 2700 cm^{-1} overlaid with the measurement field and the origin of the selected spectra (colored circles), c) PE indicator region 2685 – 2575 cm^{-1}, d) CH_2-bending at 1500 – 1400 cm^{-1}, e): CH_3 bend at 1400 – 1325 cm^{-1}, f): absolute intensity at peak position peak between at 1500 – 1400 cm^{-1}; g) absolute intensity of the peak between 1400 – 1325 cm^{-1}

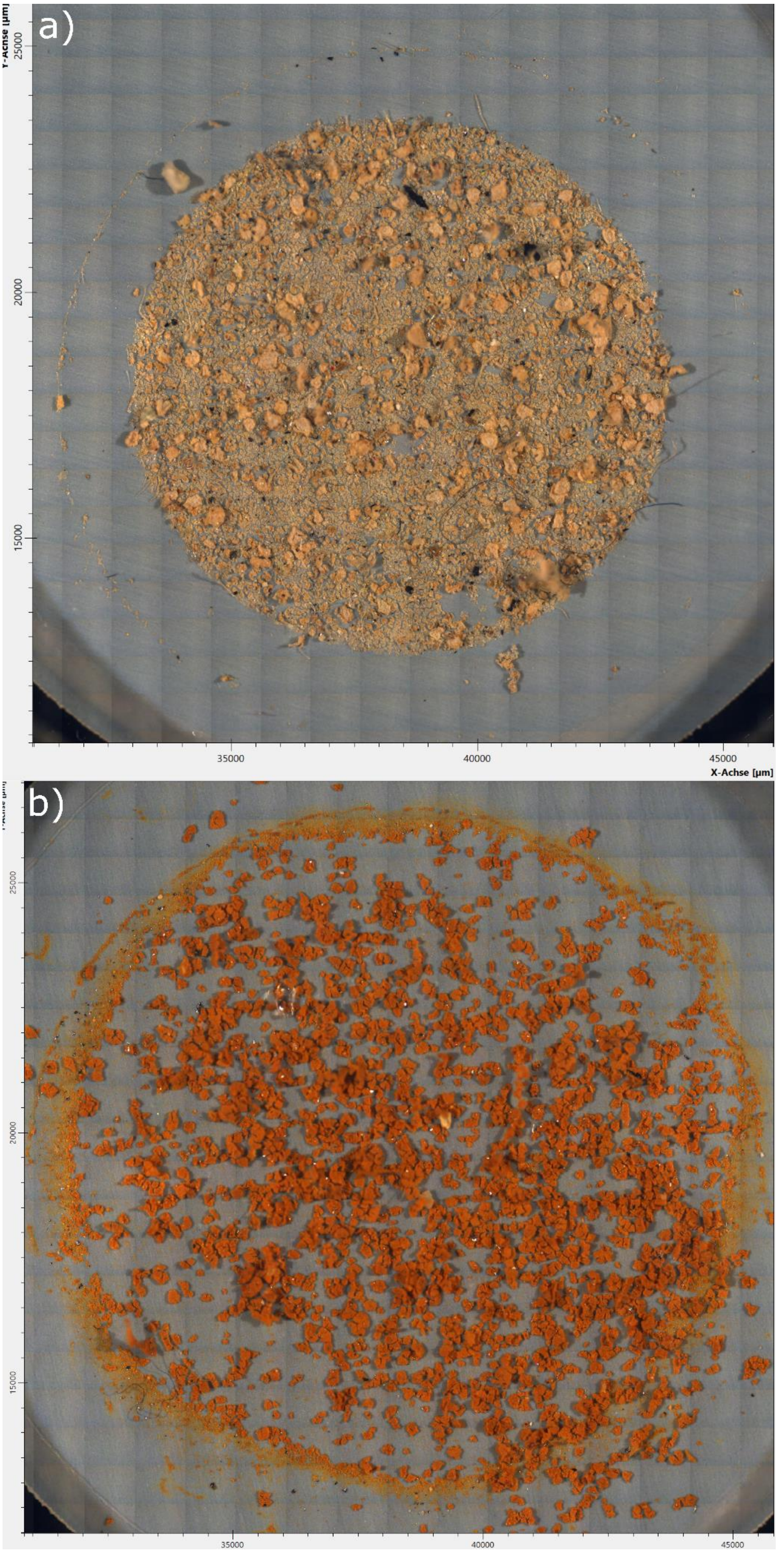

Fig. S7 Residuals on the Filter Holdorf1308 (a) and Oldenburg1308NF (b)

Paragraph S4: Mass calculation via average particle sizes based on the measured 2D area.

To take the different sizes and shapes into account of the various particles the approach of Simon et al. [9] was amended by the calculation of a representative average particle (reference particle) to add a weight to the individual particle. Here, the average particle length and minimum width were calculated for each polymer type.

For this test, the samples Oldenburg1308VF, Holdorf1708, Oldenburg1708VF were chosen due to their large deviation from the Py-GC/MS results. The mass of the reference particle was determined accordingly to Simon et al. [9] for each polymer type. The number of reference particles per polymer type was first determined by dividing the individual particle volume (V_{FTIR}) with the reference particle volume (V_{RefP}, see Equation S1), which yielded a similar result to the existing method (data not shown).

$$m_{\mathrm{Calc.,P}} = \frac{V_{\mathrm{FTIR}}}{V_{\mathrm{RefP}}} \times mass_{\mathrm{RefP}} \qquad \text{Equation S1}$$

Due to the fact, that FTIR imaging also determines the 2D area of the particles, the area ($area_{\mathrm{RefP}}$) of the reference particle (Length × Width) was determined. The measured area was divided by the reference area to determine the number of reference particles. The mass ($m_{\mathrm{Calc.,P}}$) was calculated by multiplying the number with the reference particle volume (see Equation S2). The results are depicted in Fig. 5.

$$m_{\mathrm{Calc.,P}} = \frac{area_{\mathrm{FTIR}}}{area_{\mathrm{RefP}}} \times mass_{\mathrm{RefP}} \qquad \text{Equation S2}$$

References

1. Löder MGJ, Kuczera M, Mintenig S, Lorenz C, Gerdts G. Focal plane array detector-based micro-Fourier-transform infrared imaging for the analysis of microplastics in environmental samples. Environ Chem. 2015;12(5):563-81. doi:10.1071/en14205.
2. Primpke S, Lorenz C, Rascher-Friesenhausen R, Gerdts G. An automated approach for microplastics analysis using focal plane array (FPA) FTIR microscopy and image analysis. Anal Methods. 2017;9(9):1499-511. doi:10.1039/c6ay02476a.
3. Primpke S, Wirth M, Lorenz C, Gerdts G. Reference database design for the automated analysis of microplastic samples based on Fourier transform infrared (FTIR) spectroscopy. Anal Bioanal Chem. 2018;410(21):5131-41. doi:10.1007/s00216-018-1156-x.
4. Primpke S, A. Dias P, Gerdts G. Automated identification and quantification of microfibres and microplastics. Anal Methods. 2019;11(16):2138-47. doi:10.1039/C9AY00126C.
5. Stein SE. An integrated method for spectrum extraction and compound identification from gas chromatography/mass spectrometry data. J Am Soc Mass Spectrom. 1999;10(8):770-81. doi:https://doi.org/10.1016/S1044-0305(99)00047-1.
6. Fischer M, Scholz-Böttcher BM. Simultaneous Trace Identification and Quantification of Common Types of Microplastics in Environmental Samples by Pyrolysis-Gas Chromatography-Mass Spectrometry. Environ Sci Technol. 2017;51(9):5052-60. doi:10.1021/acs.est.6b06362.
7. Fischer M, Scholz-Böttcher BM. Microplastics analysis in environmental samples – recent pyrolysis-gas chromatography-mass spectrometry method improvements to increase the reliability of mass-related data. Anal Methods. 2019;11(18):2489-97. doi:10.1039/C9AY00600A.
8. Van Den Dool H, Kratz PD. A GENERALIZATION OF THE RETENTION INDEX SYSTEM INCLUDING LINEAR TEMPERATURE PROGRAMMED GAS-LIQUID PARTITION CHROMATOGRAPHY. J Chromatogr. 1963;11:463-71.
9. Simon M, van Alst N, Vollertsen J. Quantification of microplastic mass and removal rates at wastewater treatment plants applying Focal Plane Array (FPA)-based Fourier Transform Infrared (FT-IR) imaging. Water Res. 2018;142:1-9. doi:10.1016/j.watres.2018.05.019.
